# Identification of Aging-Associated Gene Expression Signatures That Precede Intestinal Tumorigenesis

**DOI:** 10.1371/journal.pone.0162300

**Published:** 2016-09-02

**Authors:** Yoshihisa Okuchi, Masamichi Imajo, Rei Mizuno, Yuji Kamioka, Hiroyuki Miyoshi, Makoto Mark Taketo, Satoshi Nagayama, Yoshiharu Sakai, Michiyuki Matsuda

**Affiliations:** 1 Departments of Pathology and Biology of Diseases, Graduate School of Medicine, Kyoto University, Kyoto, Japan; 2 Department of Surgery, Graduate School of Medicine, Kyoto University, Kyoto, Japan; 3 Laboratory of Bioimaging and Cell Signaling, Graduate School of Biostudies, Kyoto University, Kyoto, Japan; 4 Innovative Techno-Hub for Integrated Medical Bio-Imaging, Kyoto University, Kyoto, Japan; 5 Department of Pharmacology, Graduate School of Medicine, Kyoto University, Kyoto, Japan; 6 Gastroenterological Center, Department of Gastroenterological Surgery, Cancer Institute Hospital, Japanese Foundation for Cancer Research, Tokyo, Japan; University Claude Bernard Lyon 1, FRANCE

## Abstract

Aging-associated alterations of cellular functions have been implicated in various disorders including cancers. Due to difficulties in identifying aging cells in living tissues, most studies have focused on aging-associated changes in whole tissues or certain cell pools. Thus, it remains unclear what kinds of alterations accumulate in each cell during aging. While analyzing several mouse lines expressing fluorescent proteins (FPs), we found that expression of FPs is gradually silenced in the intestinal epithelium during aging in units of single crypt composed of clonal stem cell progeny. The cells with low FP expression retained the wild-type *Apc* allele and the tissues composed of them did not exhibit any histological abnormality. Notably, the silencing of FPs was also observed in intestinal adenomas and the surrounding normal mucosae of *Apc*-mutant mice, and mediated by DNA methylation of the upstream promoter. Our genome-wide analysis then showed that the silencing of FPs reflects specific gene expression alterations during aging, and that these alterations occur in not only mouse adenomas but also human sporadic and hereditary (familial adenomatous polyposis) adenomas. Importantly, pharmacological inhibition of DNA methylation, which suppresses adenoma development in *Apc*-mutant mice, reverted the aging-associated silencing of FPs and gene expression alterations. These results identify aging-associated gene expression signatures that are heterogeneously induced by DNA methylation and precede intestinal tumorigenesis triggered by *Apc* inactivation, and suggest that pharmacological inhibition of the signature genes could be a novel strategy for the prevention and treatment of intestinal tumors.

## Introduction

Aging is one of the major risk factors for many human disorders including cardiovascular diseases [1], neurodegenerative diseases [2], diabetes [3] and cancers [4]. Indeed, aging-associated alterations of stem cell function have been implicated in many diseases. The mechanistic basis for aging-associated stem cell dysfunction has not been fully elucidated, but recent studies have implicated loss of polarity [5], mitochondrial dysfunction [6], altered autophagy [7], replication stress [8], and accrual of DNA damage [9] in stem cell aging. In addition, increasing evidence suggests that epigenetic dysregulation is also an important mechanistic driver of stem cell aging [10]. Since epigenetic alterations arising in stem cells are stably transmitted to daughter cells, they can be perpetuated and amplified within the stem cell pool via self-renewal divisions, which might have a direct functional consequence in stem cells themselves and/or their differentiated progeny and lead to decline in tissue functions. Even when the epigenetic alterations alone do not cause apparent tissue dysfunction, clones harboring the alterations might serve as the reservoir in which additional genetic alterations could arise and eventually lead to malignancy [10].

Among disorders whose incidence dramatically increases with age is colorectal cancer (CRC). Typically, sporadic CRCs develop through the adenoma-carcinoma sequence, an archetypal model of multi-step carcinogenesis, in which normal colorectal epithelium transforms to an adenoma and ultimately to an invasive/metastatic carcinoma by sequential accumulation of genetic mutations [11]. In this model, mutations of the *adenomatous polyposis coli* (*APC*) gene have been regarded as the earliest and the rate-limiting event of tumor initiation [12]. Accordingly, hereditary mutations in one of the two *APC* alleles cause familial adenomatous polyposis (FAP) that is characterized by development of a large number of colorectal adenomas [13–16]. In FAP patients, loss or inactivation of the remaining wild-type *APC* allele triggers adenoma formation. This can be recapitulated in mouse models that have hereditary mutations in one *Apc* allele [17–19]. Following *APC* mutation, a second mutation in another gene such as *KRAS* provides a growth advantage and promotes the accumulation of mutations in genes such as *PIK3CA*, *SMAD4*, and *TP53*, leading to malignant tumors [20]. Notably, several studies have reported that epigenetic changes during aging might precede the genetic changes in various cancers including colorectal cancers [21–26]. For example, it has been reported that methylation of CpG islands in a specific gene increases progressively with age in normal human colorectal mucosa and predisposes to sporadic colorectal tumorigenesis [27]. Hypo- or hypermethylation of DNA in various genes including tumor suppressor genes might also contribute to the expansion of a precursor population of colorectal tumors [28–30]. In addition, the aberrant DNA methylation profiles are often observed at the onset of colorectal tumorigenesis [24–26]. Furthermore, pharmacological inhibition of DNA methyltransferases by administration of 5-aza-2’-deoxycytidine (5-aza-dC) reduces the number of adenomas formed in the *Apc*^*Min*^ mouse model [31], while elevated methyltransferase activity induced by overexpression of *Dnmt3b* increases it [32]. These observations suggest a tumor-promoting role of the aberrant DNA methylation at the early stage of colorectal tumorigenesis. Importantly, however, most studies conducted so far focused on the epigenetic alteration in the whole tissue, not in each cell, due to technical difficulties of distinguishing aging cells from non-aging ones in living tissues. Thus, it remains unclear what genetic and/or epigenetic alterations accumulate in individual cells during aging and contribute to intestinal tumorigenesis.

We have recently generated transgenic mice expressing fluorescent protein (FP)-based biosensors for signaling molecules [33, 34]. In these mouse lines, FPs were expressed ubiquitously in the intestinal epithelium of young animals; however, we incidentally found that the expression of FPs was gradually silenced in the intestinal epithelium during aging in units of single crypts. In addition, the silencing of FPs also occurred in any of the adenomas observed in *Apc*-mutant mice and their surrounding normal mucosa. We herein show that the aging-associated silencing of FPs could be used as a biomarker of a certain fraction of cells that acquire altered gene expression signatures during aging. Our analyses then show that the aging-associated gene expression signatures are induced, at least in part, by DNA methylation and precede intestinal tumorigenesis triggered by *Apc* inactivation.

## Materials and Methods

### Mice

Transgenic mice expressing the Förster resonance energy transfer (FRET) biosensor for the extracellular signal-regulated kinase (ERK) (EKAREV mice) or the protein kinase A (PKA) (AKAREV mice) have been described previously [34]. Founder animals were backcrossed more than ten generations to C57BL/6N Jcl (CLEA Japan, Tokyo, Japan). To date, no disease or anomaly has been observed in these mice. Transgenic mice expressing the enhanced GFP (EGFP) (Green mice) [35] were obtained from Japan SLC (Shizuoka, Japan). In all the transgenic mice described above, the transgenes (fluorescent proteins) were expressed under the control of the CAG promoter [36]. *Apc*^*Δ716*^ mice have been reported previously [19]. R26-H2B-mCherry mice were obtained from RIKEN CDB [37]. Mice were housed in a specific pathogen-free facility and fed with a standard diet and water ad libitum. In some experiments, twenty-six-week-old EKAREV mice were daily administered subcutaneously with either 5-aza-dC (1 mg/kg body weight; Sigma-Aldrich, St Louis, MO, USA) or vehicle (phosphate-buffered saline, PBS) for two weeks. The animal protocols have been reviewed and approved by the Animal Care and Use Committee of Kyoto University Graduate School of Medicine (No. 10584).

### Human FAP samples

Human colorectal specimens were resected from four FAP patients during total or subtotal colectomy at the Cancer Institute Hospital of the Japanese Foundation for Cancer Research. We collected adenomas, the surrounding normal mucosae (within 5 mm from the adenomas), and the mucosae located more than 2 cm away from the adenomas. Mucosae were carefully dissected from the muscular layer with scissors. Thus, most of the collected cells should be epithelial cells. However, a small number of cells other than epithelial cells might be included in the samples. This study protocol was approved by the Institutional Review Boards of Cancer Institute Hospital (2011–1075) and Kyoto University (G630) and patients consented to the use and analysis of their tissue samples in writing.

### Microscopy and image processing

For two-photon excitation microscopy (2PM), we used an FV1200MPE-IX83 inverted microscope (Olympus, Tokyo, Japan) equipped with a 30x/1.05 NA silicon oil-immersion objective lens (UPLSAPO 30XS; Olympus) and an FV1200MPE-BX61WI upright microscope equipped with a 25x/1.05 water-immersion objective lens (XLPLN 25XWMP; Olympus) and an InSight DeepSee Laser (Spectra Physics, Santa Clara, CA, USA). The laser power was set to 8–10% and 2–4% for the observation of the intestine and the skin, respectively [33, 38]. The scan speed was set at 20 μs/pixel. We used 840-nm light to excite CFP. We used an IR-cut filter (BA685RIF-3), two dichroic mirrors (DM505 and DM570), and two emission filters (BA460-500 for CFP and BA520-560 for YFP) (Olympus). Acquired images were analyzed with MetaMorph software (Universal Imaging, West Chester, PA, USA) as described previously [34, 39].

Confocal images were acquired with an FV1000/IX83 confocal microscope (Olympus) equipped with a 30x/1.05 NA silicon oil-immersion objective lens (UPLSAPO 30XS; Olympus).

### Quantification of crypts expressing fluorescent proteins

The small intestine was resected, opened longitudinally and washed with cold PBS. The tissues were then observed by 2PM. In each tissue sample, we randomly observed at least five view fields, and in each field we quantified the percentage of the crypts in which epithelial cells expressed fluorescent proteins. In some crypts, only Paneth cells expressed the fluorescent proteins. These crypts were classified as “non-fluorescent” crypts.

### Immunofluorescent staining

Small intestine specimens were washed with PBS and fixed overnight in 10% formalin in PBS at 4°C. After fixation, tissues were embedded in O.C.T. Compound (Tissue-Tek: Sakura Finetek Japan, Tokyo, Japan), frozen, and sectioned at 5 μm thickness. Sections were directly observed with a fluorescent microscope or subjected to immunofluorescent staining with an antibody against GFP (Clontech, Mountain View, CA, USA). Alexa Fluor 488 goat anti-rabbit IgG antibody (Thermo Fisher Scientific, Waltham, MA, USA) was used as the secondary antibody. Counterstaining was performed with Hoechst33342 (Thermo Fisher Scientific). To stain secretory granules in Paneth cells and goblet cells, we used Rhodamine-labeled Ulex Europaeus Agglutinin I (UEA I) (Vector Laboratories, Burlingame, CA, USA). For immunofluorescent staining of GFP, antigen retrieval was performed by boiling samples for 30 min in Tris-HCl buffer pH 8.0 containing 1 mM EDTA.

### Isolation of FP^lo^ and FP^hi^ cells by FACS

Intestinal crypts were isolated from the mouse small intestine by incubation for 30 min at 4°C in PBS containing 2 mM EDTA. The isolated intestinal crypts were then dissociated into single cells by incubation with 0.25% trypsin at 37°C for 1 min, followed by vigorous pipetting. Cells were filtered with a 40 μm cell strainer (BD Falcon, Franklin Lakes, NJ, USA) and sorted by a FACSAria II cell sorter (BD), depending on the expression levels of the fluorescent biosensor (EKAREV). We termed the cells with low and high FP expression FP^lo^ and FP^hi^ cells, respectively. The sorted cells were subjected to an intestinal organoid culture or DNA/RNA analysis.

### Intestinal organoid culture

The intestinal epithelial cells were isolated as described above, embedded in Matrigel (Thermo Fisher Scientific), and cultured in the L-WRN cell-conditioned medium supplemented with 10 μM Y-27632, a ROCK inhibitor (R&D Systems, Minneapolis, MN, USA), and 10 μM SB431542, an inhibitor of the TGF-β type I receptor (R&D Systems), as described previously [40]. Adenoma cells were isolated from the *Apc*^*Δ716*^ mouse small intestinal adenoma by incubation for 60 min at 4°C in PBS containing 2 mM EDTA, embedded in Matrigel, and cultured in Advanced DMEM/F12 supplemented with 20% FBS, Y-27632 and SB431542. In some experiments, 10 μM 5-aza-dC or vehicle (DMSO) was added into the medium one day after passage.

### Extraction of DNA and RNA, qRT-PCR, and genomic PCR

DNA and RNA were extracted by using NucleoSpin RNA (TaKaRa, Shiga, Japan) and a NucleoSpin RNA/DNA Buffer Set (TaKaRa) according to the manufacturer’s protocol. The extracted total RNA was reverse-transcribed into cDNA by using a High-Capacity cDNA Reverse Transcription Kit (Thermo Fisher Scientific). Quantitative PCR analyses were performed by using an Applied Biosystems StepOne Real-Time PCR System and Power SYBR Green PCR Master Mix (Thermo Fisher Scientific). The expression data obtained for each gene were normalized to those of GAPDH or the average data of five housekeeping genes: GAPDH, ALAS1, RPS18, B2M and HPRT1. The extracted DNA was analyzed by genomic PCR to quantify the ratio of the wild-type *Apc* to the *Apc*^*Δ716*^ alleles.

### Bisulphite genomic sequencing

Methylation of genomic DNA was analyzed by DNA bisulphite sequencing [41] using a MethylEasy Exceed Rapid DNA Bisulphite Modification Kit (Human Genetic Signatures, Sydney, NSW, Australia). The modified DNA was PCR amplified by using TaKaRa EpiTaq HS (TaKaRa) with a primer set targeting the CAG promoter (5’-GGATTTTTTTTGTTTTAAATTTGTG-3’ and 5’-ATAATAAAACAACACAATAACCAACA-3’) and sequenced to identify the methylated nucleotides.

### Microarray analysis

The mouse intestinal crypts were isolated as described above. Under fluorescent microscopic observation, we picked up crypts that expressed (FP^hi^) or did not express (FP^lo^) fluorescent protein by using 40 μm-diameter glass capillary pipettes. RNAs were extracted by using NucleoSpin RNA XS (TaKaRa). The quality of RNA was examined with an Agilent 2100 bioanalyzer and RNA Pico LabChip kits (Agilent Technologies, Santa Clara, CA, USA). The RNA Integrity Numbers (RINs) of RNA samples used in microarray analysis were above 7. RNA samples were amplified by using an Ovation One-Direct System (NuGEN, San Carlos, CA, USA), labeled with the Encore Biotin Module (NuGEN), and hybridized to the Mouse Gene 1.0 ST arrays (Affymetrix, Santa Clara, CA, USA). For microarray analysis of human FAP samples, total RNA was extracted by using an RNeasy Mini Kit (QIAGEN, Valencia, CA, USA). Synthesis of cDNA, *in vitro* transcription and biotin labeling of cRNA, and hybridization to the Human Gene 1.0 ST arrays (Affymetrix) were performed by using the Ambion WT Expression Kit (Thermo Fisher Scientific) according to the manufacturer’s protocol. Hybridized arrays were scanned using an Affymetrix GeneChip Scanner. The obtained data were analyzed by using GeneChip Command Console, Expression Console, and Transcriptome Analysis Console software. Gene ontology analysis was performed by using the DAVID bioinformatics resources (https://david.ncifcrf.gov).

### ELISA assay

The urinary F2-isoprostane concentration, the plasma IL-6 concentration and the concentration of PGE2 secreted from intestinal epithelial cells were measured by using commercially available ELISA kits; Urinary Isoprostane EIA kit (Oxford Biomedical Research, Oxford, MI, USA), Murine IL-6 ELISA Kit (Diaclone SAS, Besancon, France) and PGE2 high sensitivity ELISA kit (Enzo Life Sciences, Farmingdale, NY, USA). Each experiment was performed according to the manufacturer’s protocols.

### Statistical analysis

When the data followed normal distribution, we used the Student’s *t*-test to examine statistical significance. Fisher’s exact test was used to determine whether there were nonrandom associations between two gene sets identified from mice and human microarray data.

## Results

### Loss of fluorescent protein expression in the histologically-normal epithelium of aging mice

**Fig 1:**
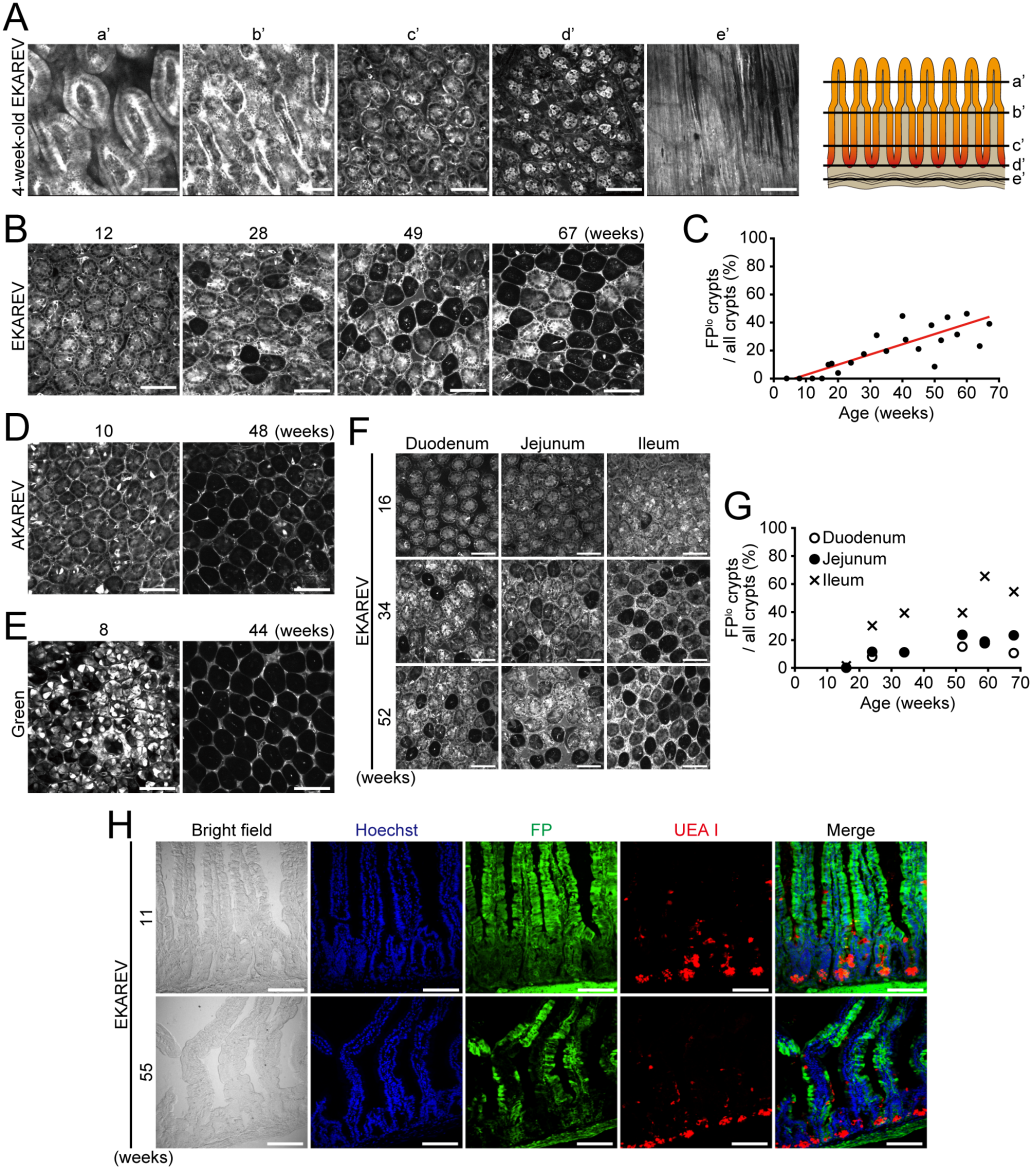
Aging-associated loss of fluorescent protein expression in the normal intestinal mucosa. (A) Expression of CFP in the small intestine of a 4-week-old EKAREV mouse was observed by 2PM. The planes shown in each picture are indicated in a schematic illustration (right): (a’-e’) planes crossing villi (a’), transition region between villi and crypts (b’), middle (c’) and bottom regions of crypts (d’), and the smooth muscle layer (e’). Scale bars, 100 μm. (B) Representative images showing the distribution of CFP in the small intestine (jejunum) of 12-, 28-, 49- and 67-week-old EKAREV mice. The plane crossing middle region of the crypt was observed by 2PM. Scale bars, 100 μm. (C) Percentage of FP^lo^ crypts among all crypts in the small intestine (jejunum) of the EKAREV mice. A red line indicates the fitting result. Note that there was a linear correlation between aging and the ratio of FP^lo^ crypts to all crypts. (D and E) Representative CFP images of intestinal (jejunum) crypts in a 10-week-old (left) or 48-week-old (right) AKAREV mouse (D) and an 8-week-old (left) or 44-week-old (right) Green mouse (E) taken by 2PM. Scale bars, 100 μm. (F) Representative CFP images of the duodenum (left), jejunum (middle) and ileum (right) of 16- (top), 34- (middle) and 52-week-old (bottom) EKAREV mice taken by 2PM. Scale bars, 100 μm. (G) Percentage of FP^lo^ crypts among all crypts in each region of the small intestine at different ages. (H) Sections of the young (11-week-old) or aged (55-week-old) EKAREV mouse small intestine were observed with a confocal microscope. The nuclei of cells and secretory granules in goblet and Paneth cells were stained with Hoechst (blue) and rhodamine-labeled UEA I (red), respectively. Expression of the biosensor was directly observed without staining (FP, green). Scale bars, 100 μm.

We have recently established transgenic mouse lines that stably express Förster resonance energy transfer (FRET) biosensors for the extracellular signal-regulated kinase (ERK) (EKAREV mice) and the protein kinase A (PKA) (AKAREV mice) [34]. We observed these mice by two-photon excitation microscopy (2PM) and showed that these mice are useful to visualize the ERK and PKA activities in many tissues including the intestinal epithelium [33, 34]. During the observation, we incidentally noticed aging-associated loss of FPs in the intestinal epithelium; FPs were ubiquitously expressed in the intestinal epithelium of young EKAREV mice (Fig 1A), but its expression was lost in a fraction of crypts in aging EKAREV mice (Fig 1B). The crypts composed of cells with low FP expression, which was termed FP^lo^ cells, appeared around 16 weeks and gradually increased with age (Fig 1B and 1C). Similar phenomena were observed in AKAREV mice [34] and the mice expressing enhanced GFP (EGFP) (Green mice) [35] (Fig 1D and 1E). The percentage of the crypts composed of FP^lo^ cells was also increased along the direction from the duodenum to the ileum (Fig 1F and 1G). Immunofluorescent staining confirmed that FPs were expressed in most epithelial cells of young EKAREV mice, but not in a significant fraction of epithelial cells of aging EKAREV mice (Fig 1H). Notably, the epithelium composed of FP^lo^ cells was morphologically normal and indistinguishable from that of cells with high FP expression (FP^hi^ cells) (Fig 1H). In addition, distribution of goblet cells and Paneth cells, which were stained by the UEA I staining, was similar between the tissues composed of FP^lo^ and FP^hi^ cells (Fig 1H). These results show that expression of the FPs is gradually lost in units of single crypt during aging and that the loss of FPs itself is not associated with any histological alterations.

We also examined whether the aging-associated loss of FPs is specific to the intestine or common among different tissues. To this end, we investigated the expression status of FPs in the hematopoietic system and the skin, which are constantly renewed from stem cells as in the case of the intestinal epithelium. However, expression of FPs was not silenced in these tissues during aging (S1 and S2 Figs). Thus, as far as we investigated, the loss of FPs is specific to the intestinal epithelium.

### Loss of fluorescent protein expression in and around adenomas

**Fig 2:**
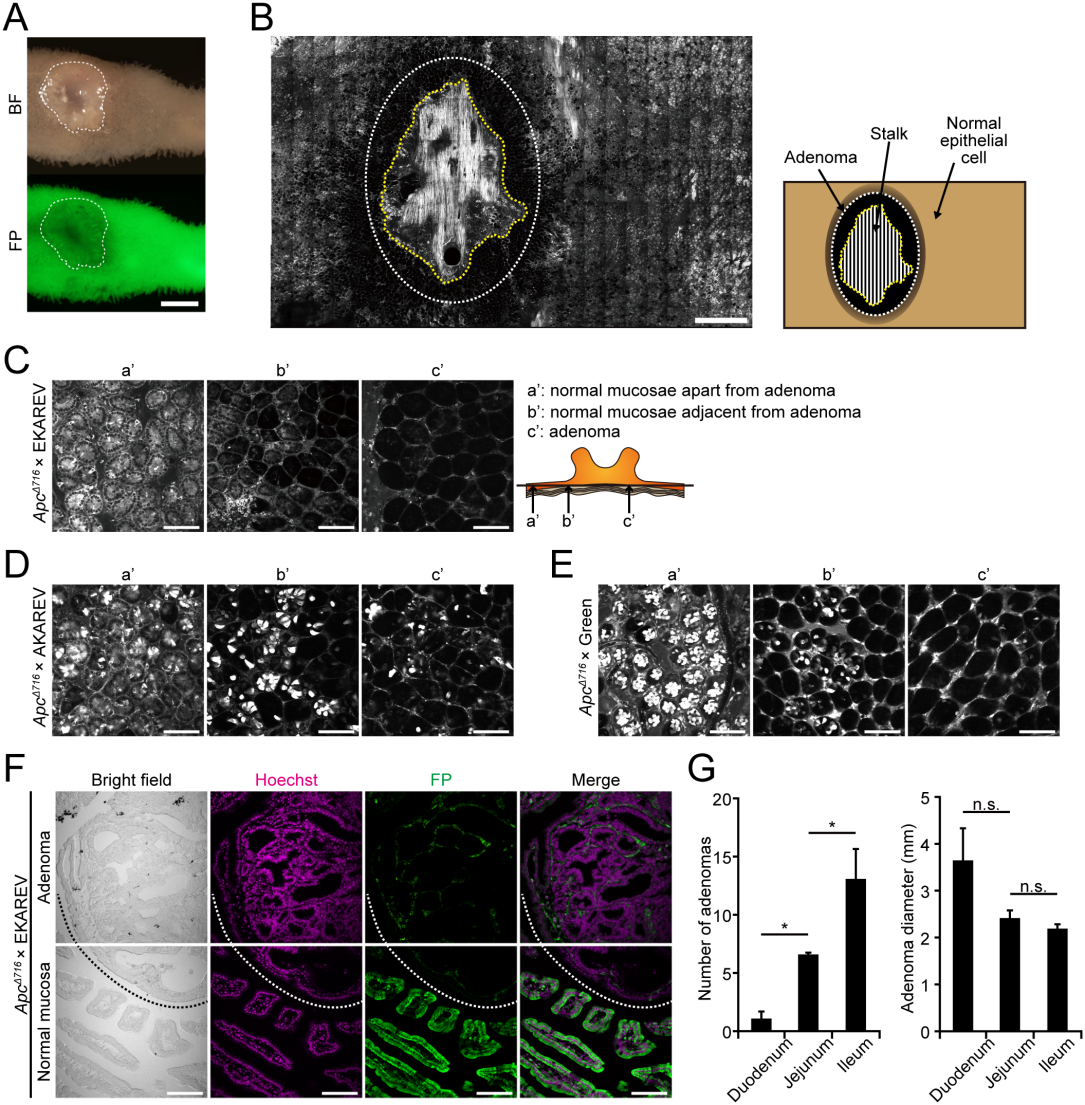
Loss of fluorescent protein expression in intestinal adenomas and the neighboring normal mucosae of *Apc*^*Δ716*^ mice. (A) Representative macroscopic photographs of an adenoma and the surrounding normal tissue in the duodenum of a 22-week-old *Apc*^*Δ716*^ × EKAREV mouse taken by a fluorescence stereo microscope. BF, Bright field (upper); FP, CFP image (lower). White dotted lines indicate the boundary between the adenoma and the normal tissue. Scale bar, 1 mm. (B) Distribution of the ERK biosensor in the intestinal (jejunum) mucosa of a 29-week-old *Apc*^*Δ716*^ × EKAREV mouse. The image was made by combining 14 × 23 CFP images of intestinal crypts taken by 2PM. A white dotted ellipse indicates the approximate size and position of an adenoma. The region encircled by a yellow dotted line corresponds to the stalk of the adenoma, in which thickened interstitial tissues are observed. Scale bar, 1 mm. (C-E) Representative CFP (C and D) and EGFP (E) images of intestinal crypts located in and around adenomas: normal mucosae apart from adenomas (a’), normal mucosae adjacent to adenomas (b’) and adenomas (c’) of *Apc*^*Δ716*^ × EKAREV (C), *Apc*^*Δ716*^ × AKAREV (D) and *Apc*^*Δ716*^ × Green mice (E). Images shown in (C) are magnified views of a part of the low-magnification image shown in (B). Note that EGFP is distributed throughout cells in Green mice, whereas the fluorescent proteins are localized to the cytoplasm in EKAREV and AKAREV mice. Scale bars, 100 μm. (F) Immunofluorescent staining of an adenoma and the surrounding normal tissue of an *Apc*^*Δ716*^ × EKAREV mouse with an anti-GFP antibody (FP, green). The nuclei were stained with Hoechst (magenta). Scale bars, 100 μm. (G) Number (left) and size (right) of intestinal tumors formed in *Apc*^*Δ716*^ × EKAREV mice (mean ± SE, n = 4: 2 males and 2 females). *P < 0.05; n.s., not significant (Student’s *t*-test).

Next, we inquired the expression status of FPs in intestinal adenomas developed in the EKAREV mice crossed with *Apc*^*Δ716*^ mice (*Apc*^*Δ716*^), which have a truncation mutation in the *Apc* gene and serve as a model of human familial adenomatous polyposis (FAP) [19], as colorectal tumor incidence significantly increases with age. Expression of FPs was almost completely lost in the adenoma cells (Fig 2A–2C). Subsequent analysis also revealed that the loss of FP was not restricted to adenoma cells but also frequently observed in the epithelial cells adjacent to the adenomas (Fig 2C). Indeed, the number of FP^lo^ cells was apparently increased in the epithelium adjacent to the adenomas compared to that apart from the adenomas (Fig 2C). We also observed intestinal adenomas of AKAREV or Green mice crossed with the *Apc*^*Δ716*^ mice. Similarly to the *Apc*^*Δ716*^ × EKAREV mice, expression of FPs was lost in the adenoma cells and in the majority of the epithelial cells adjacent to the adenomas in these mice (Fig 2D and 2E). Again, immunofluorescent staining confirmed the loss of FPs in and around adenomas (Fig 2F). Importantly, we did not observe significant differences in the number and location of adenomas between the heterozygous *Apc*^*Δ716*^ mice and the *Apc*^*Δ716*^ × EKAREV mice, negating the effect of the biosensor expression on the development of adenomas. As reported previously in the heterozygous *Apc*^*Δ716*^ mice [42], the number of adenomas in the small intestine of *Apc*^*Δ716*^ × EKAREV mice was increased along the direction from the duodenum to the ileum, but the size of adenomas did not change significantly in any regions (Fig 2G). As the percentage of FP^lo^ cells was also increased along the duodenum-ileum axis (Fig 1F and 1G), the incidence of FP^lo^ cells correlated well with that of adenomas. We thus hypothesized that the FP^lo^ cells are involved in the early stages of intestinal tumorigenesis in the *Apc*-mutant mice.

### Hypermethylation of the CAG promoter in adenoma cells

**Fig 3:**
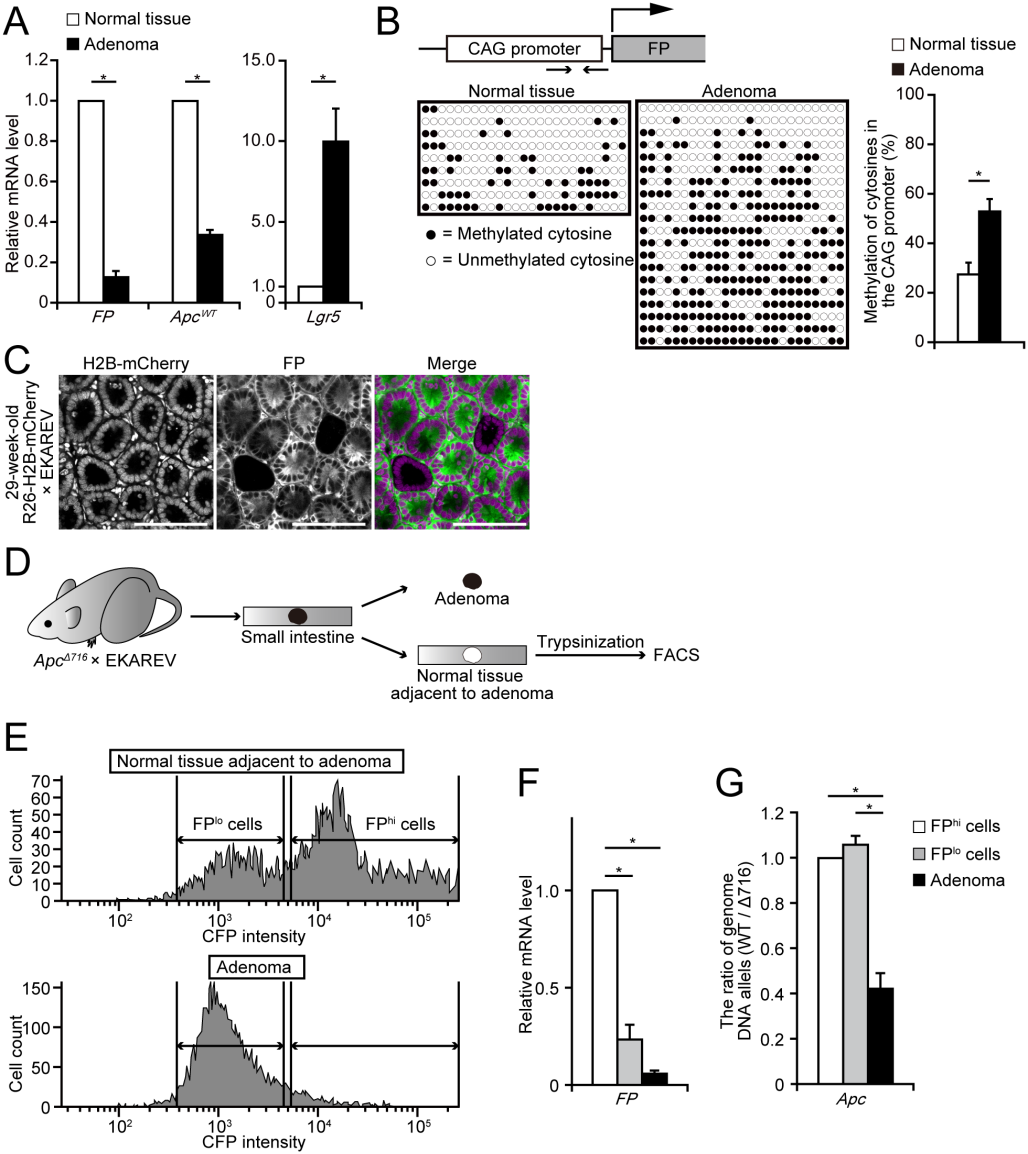
Hypermethylation of the CAG promoter in FP^lo^ and adenoma cells. (A) Relative mRNA expression levels of *FP*, *Apc*^*WT*^, and *Lgr5* in the normal intestinal mucosae of EKAREV mice and adenomas of *Apc*^*Δ716*^ × EKAREV mice were analyzed by quantitative RT-PCR (n = 3 littermates). *P < 0.05 (Student’s *t*-test). (B) Bisulphite sequencing analyses on the methylation status of the CAG promoter in the normal intestinal mucosae of EKAREV mice and adenomas of *Apc*^*Δ716*^ × EKAREV mice. (upper) Scheme showing the structure of the EKAREV expression cassettes. Arrows indicate the approximate position of primers used in the analyses. (lower) Panels show the methylation status of 23 CpGs, which were located in the analyzed region of the CAG promoter, in 9 samples from the normal intestinal mucosa of an EKAREV mouse (left) and 20 samples from adenomas of an *Apc*^*Δ716*^ × EKAREV mouse (right). Each white or black circle means an unmethylated or a methylated CpG site, respectively. The bar graph shows the quantification of the results (mean ± SE). (C) Representative images showing expression of H2B-mCherry (magenta) in the nucleus and that of EKAREV (FP, green) in the cytoplasm in a 29-week-old R26-H2B-mCherry × EKAREV mouse. Note that, in some crypts, the expression of EKAREV, but not that of H2B-mCherry, was lost (silenced). Scale bars, 100 μm. (D) Scheme showing the procedures performed in FACS analysis of adenomas and the adjacent normal tissues. (E) Histograms of CFP fluorescence intensity obtained from FACS analysis of adenomas and the adjacent normal tissues. (F and G) Relative mRNA expression levels of *FP* (F) and the ratio of *Apc*^*WT*^ alleles to *Apc*^*Δ716*^ alleles (G) in FP^hi^, FP^lo^, and adenoma cells. The RNA and DNA samples analyzed here were extracted from the same samples (n = 6 for each). *P < 0.05 (Student’s *t*-test).

To address a mechanism underlying the loss of FPs in adenoma cells, we investigated the mRNA levels of FPs in normal epithelial cells and adenoma cells of *Apc*^*Δ716*^ × EKAREV mice. As expected, the mRNA level of the wild-type *Apc* (*Apc*^*WT*^) was significantly decreased, whereas that of *Lgr5*, an established intestinal stem cell marker, was increased in adenoma cells (Fig 3A), suggesting successful isolation of adenoma cells and normal cells. Notably, the mRNA level of FP was markedly decreased in the adenoma cells (Fig 3A). This suggests that expression of FPs is suppressed at the transcriptional level in adenoma cells. Since stable silencing of genes is often mediated by hypermethylation of CpG islands in the promoter regions [23], we examined the methylation status of the CAG promoter that drives expression of FPs in all the transgenic mouse lines used in this study (EKAREV, AKAREV, and Green mice) [36]. In agreement with the decreased mRNA expression of FPs, the CAG promoter was highly methylated in adenoma cells (Fig 3B). These results suggest that the loss of FPs in adenoma cells is mediated by hypermethylation of the upstream CAG promoters.

We then asked whether the silencing was specific to the CAG promoter. To this end, we crossed EKAREV mice with R26-H2B-mCherry mice, which express nuclear FPs, H2B-mCherry under the ubiquitous Rosa26 transcriptional machinery. In these mice, the expression of H2B-mCherry was not silenced during aging: H2B-mCherry was expressed in the crypts that had lost the expression of EKAREV (Fig 3C). Thus, the CAG promoter is susceptible to epigenetic silencing during aging. It has been shown that the methylation-free status of a transgene is progressively lost with increasing transgene copy number [43] and that transgene repeats cause heterochromatin formation and gene silencing [44]. To exclude these possibilities, we quantified the DNA copy numbers of the EKAREV and H2B-mCherry transgenes by qPCR (S3 Fig). The copy number of EKAREV gene was very similar to that of H2B-mCherry gene, which is inserted into the Rosa26 locus in a single copy by homologous recombination [37]. Therefore, the aforementioned mechanism of silencing may not be responsible for the silencing of the transgenes in this study.

It is known that, in the heterozygous *Apc*^*Δ716*^ mice, loss of the remaining wild-type *Apc* allele occurs exclusively by loss-of-heterozygosity (LOH), at least, on the C57BL/6 genetic background [45, 46]. We examined whether the wild-type *Apc* allele had been already deleted by LOH in the FP^lo^ cells. For this purpose, we separated FP^hi^ and FP^lo^ cells by fluorescence activated cell sorting (FACS), and compared the copy number of the wild-type *Apc* allele vs the *Apc*^*Δ716*^ allele (Fig 3D–3G). As anticipated, the expression of FPs was markedly suppressed in the FP^lo^ cells as in adenoma cells (Fig 3F). Notably, however, LOH of the *Apc* gene was not observed in FP^lo^ or FP^hi^ cells; both of FP^lo^ and FP^hi^ cells harbor the similar numbers of the wild-type *Apc* and the *Apc*^*Δ716*^ alleles (Fig 3G). In contrast, adenoma cells harbored the increased number of *Apc*^*Δ716*^ alleles, suggesting that the *Apc* LOH had already occurred in these cells. Thus, it is likely that, during the adenoma development in *Apc*^*Δ716*^ mice, the silencing of FPs caused by hypermethylation of the CAG promoters precedes LOH of the *Apc* gene.

### Gene expression profiling of FP^hi^, FP^lo^, and adenoma cells

**Fig 4:**
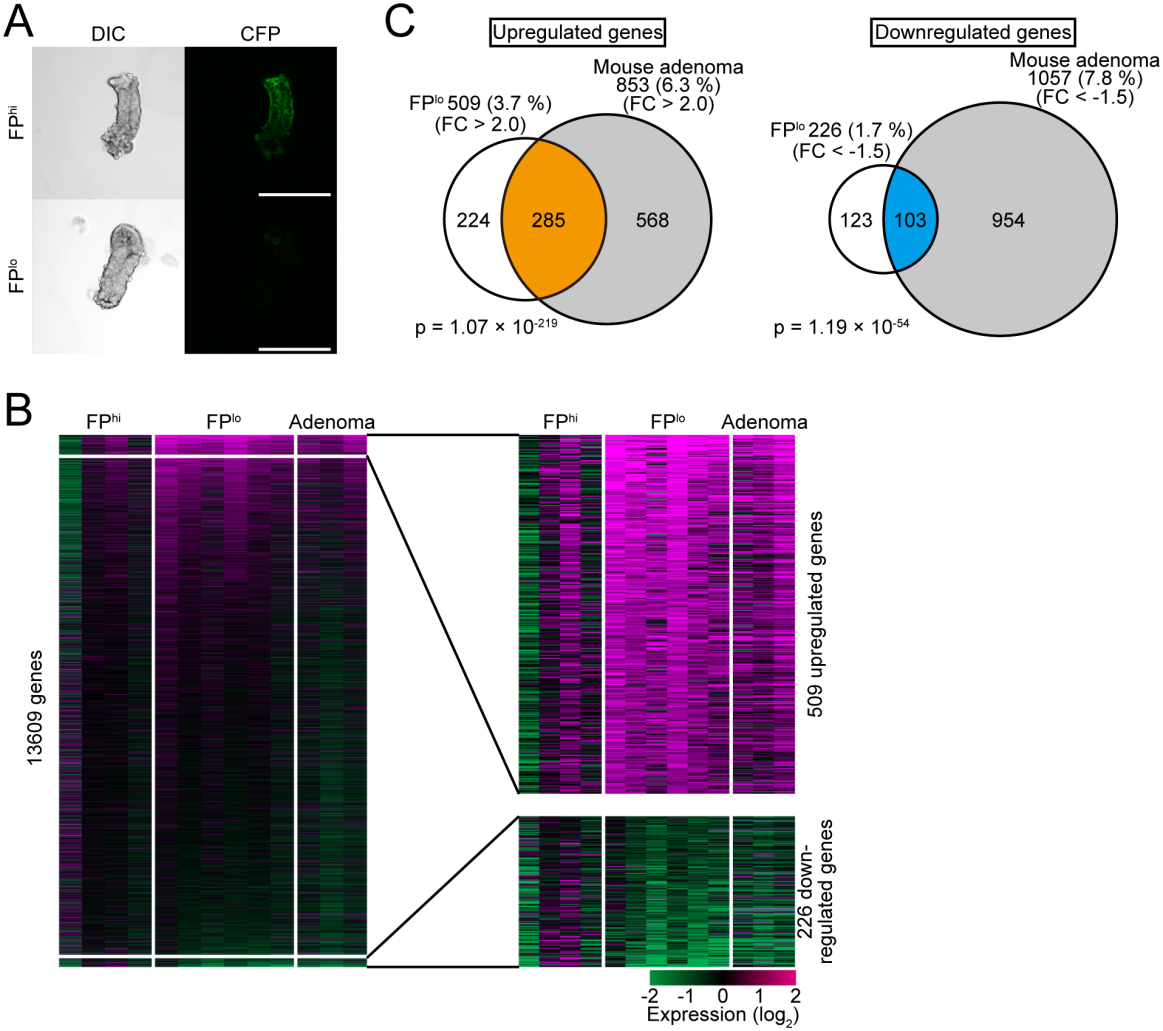
Gene expression profiling of FP^hi^, FP^lo^ and adenoma cells. (A) Representative images of FP^hi^ (top) and FP^lo^ (bottom) crypts isolated from 31-week-old *Apc*^*Δ716*^ × EKAREV mice taken with a confocal microscope. Scale bars, 100 μm. (B) Gene expression profiling of FP^hi^, FP^lo^ and adenoma cells by microarrays. Each horizontal line in the heat maps shows the expression data for one gene. FP^hi^ cells, left 4 columns; FP^lo^ cells, middle 6 columns; adenoma cells, right 3 columns. We focused on a total of 13,609 genes against which probes were designed in both mouse and human microarrays. Genes upregulated or downregulated in FP^lo^ cells were defined as those genes whose expression levels were significantly increased by 2.0-fold or decreased by 1.5-fold, respectively, compared with those in FP^hi^ cells. Expression profiles of genes up- and downregulated in FP^lo^ cells are magnified in the right heat map. (C) Venn diagrams showing statistically significant overlap between genes up- or downregulated in FP^lo^ and adenoma cells. Here, genes up- or downregulated in adenoma cells were defined as those genes whose expression levels were increased by 2.0-fold or decreased by 1.5-fold compared with those in FP^hi^ cells. *P* values were calculated by Fisher’s exact test.

We then performed gene expression profiling of FP^hi^, FP^lo^, and adenoma cells from 31-week-old *Apc*^*Δ716*^ × EKAREV to examine whether the silencing of FPs reflects some changes in the expression status of endogenous genes. Single crypts composed of either FP^hi^ or FP^lo^ cells were dissociated from the tissue by treatment with EDTA and collected with a glass capillary pipette under a fluorescent microscope (Fig 4A). From the isolated single crypts, RNA was extracted and subjected to microarray analyses. Crypt-like cell clusters derived from adenomas were similarly processed. We identified 509 and 226 genes whose expression levels were upregulated by more than 2.0-fold and downregulated by more than 1.5-fold, respectively, in FP^lo^ crypts in comparison to FP^hi^ crypts (Fig 4B). Thus, the silencing of FPs was indeed accompanied by altered expression of endogenous genes. The following gene ontology analysis showed that genes related to cell cycle progression were significantly enriched in the genes upregulated in FP^lo^ crypts (S1 Table). We then identified 853 and 1057 genes, whose expression levels were upregulated (> 2.0-fold) and downregulated (< -1.5-fold), respectively, in adenomas compared to FP^hi^ crypts. Notably, the genes upregulated and downregulated in adenomas significantly overlapped with those genes upregulated and downregulated in FP^lo^ crypts, respectively (Fig 4C). These results show that the aging-associated silencing of FPs reflects specific gene expression alterations that are also observed in adenomas.

### Gene expression profiling of adenomas and the normal mucosae adjacent to and apart from the adenomas in human patients

**Fig 5:**
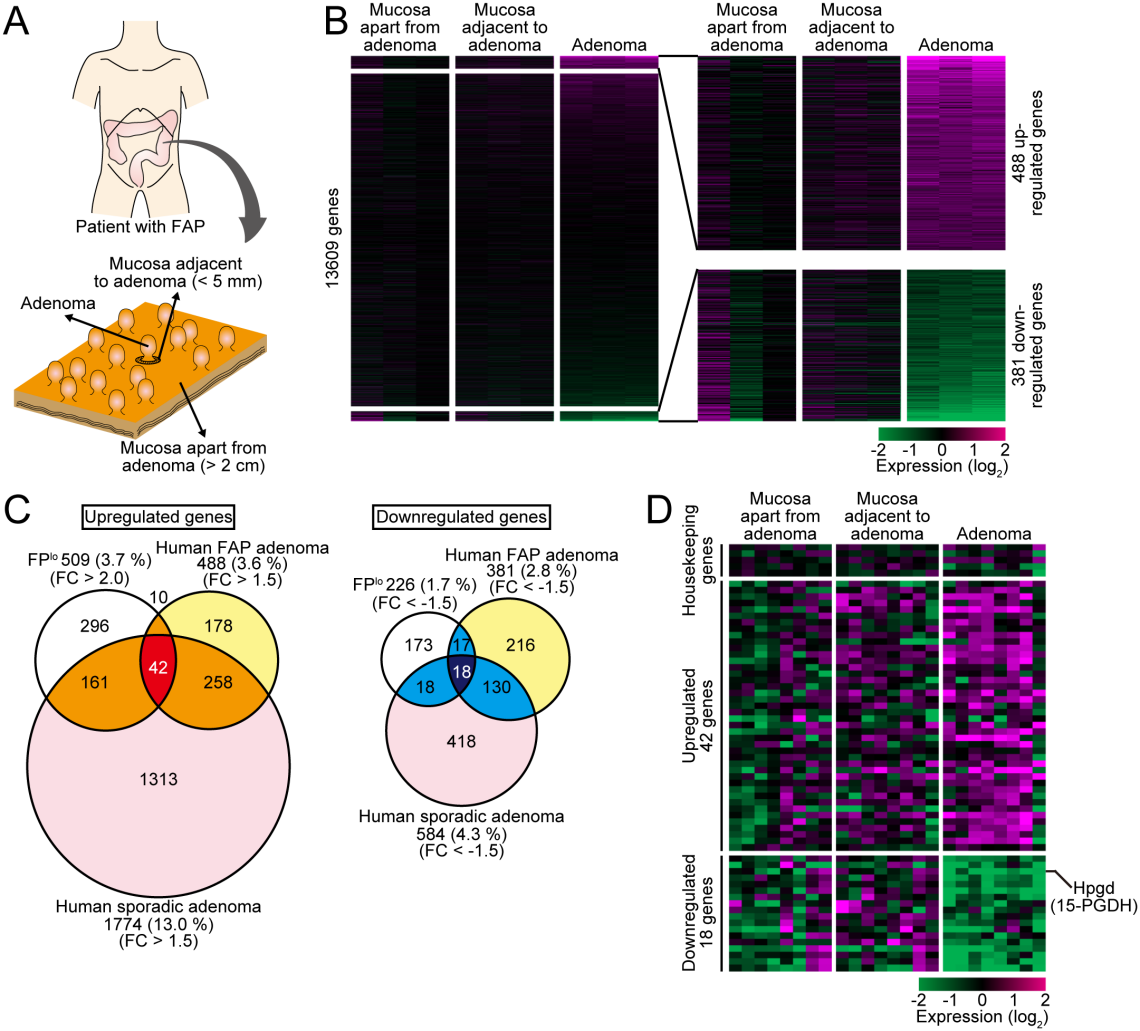
Gene expression profiling of human FAP and sporadic adenoma patient samples. (A) Schematic representation of the classification of FAP patient samples. Adenomas (5 to 10 mm in size), mucosae within 5 mm from the stalk of the adenomas (“mucosa adjacent to adenomas”), and mucosae more than 20 mm away from the adenomas (“mucosa apart from adenomas”) were resected from four Japanese FAP patients and subjected to microarray analysis. (B) Gene expression profiles of the mucosa apart from adenomas, the mucosae adjacent to adenomas, and adenomas (n = 3 specimens from one patient for each category). The left heat map shows expression profiles of a total of 13,609 genes. The right enlarged heat map shows expression profiles of genes up- or downregulated in adenomas. Note that there were no significant differences between the mucosae apart from adenomas and the mucosae adjacent to adenomas. Here, genes up- or downregulated in adenomas were defined as those genes whose expression levels were increased by 1.5-fold or decreased by 1.5-fold, respectively, compared with those in the mucosae apart from adenomas. (C) Venn diagrams comparing the gene expression profiles of FP^lo^ cells (top left), human FAP adenomas (top right) and human sporadic adenomas (bottom). We defined genes up- or downregulated in human sporadic adenomas as those genes whose expression levels were increased by 1.5-fold or decreased by 1.5-fold, respectively, compared with those in the normal mucosae. (D) Quantitative RT-PCR analysis of 5 housekeeping genes, and the 42 and 18 genes whose expression levels were commonly upregulated or downregulated, respectively, in FP^lo^ cells, human FAP adenomas, and human sporadic adenomas in the microarray analyses.

To examine the clinical relevance of our findings, gene expression analysis was performed with intestinal epithelial cells obtained from surgical specimens of four Japanese FAP patients. We compared gene expression patterns in samples collected from adenomas sized from 5 to 10 mm, mucosae within 5 mm from the stalks of adenomas, and mucosae at least 20 mm distant from adenomas (Fig 5A). The results showed that, in comparison to the mucosae apart from adenomas, 488 genes were upregulated by more than 1.5-fold and 381 genes were downregulated by more than 1.5-fold in the adenomas (Fig 5B). Notably, the expression levels of *LGR5* in adenoma samples were significantly higher than those in normal mucosae (S4 Fig), suggesting that adenoma cells were successfully separated from normal cells. The genes upregulated and downregulated in FAP adenomas significantly overlapped with those in FP^lo^ cells (Fig 5C). We also analyzed previously published datasets of human sporadic adenomas [GSE8671 [47]], and found that the 1,774 genes upregulated (> 1.5-fold) and 584 genes downregulated (< -1.5-fold) in human sporadic adenomas also significantly overlapped with those in FP^lo^ cells (Fig 5C). These results suggest that the aging-associated gene expression signatures identified in FP^lo^ cells are characteristics common to human FAP and sporadic adenomas. Notably, we identified 42 genes commonly upregulated in FP^lo^ crypts, human FAP adenomas and human sporadic adenomas (Fig 5C and S2 Table). Similarly, 18 genes commonly downregulated were identified (Fig 5C and S3 Table). About half of the identified upregulated genes (24/42 genes) have been shown to play a role in tumor development and progression (e.g. *Cd44*, *Myc*, etc.) (S2 Table). As for these 42 upregulated genes and 18 downregulated genes, qRT-PCR was performed with RNA samples obtained from the adenomas and the mucosae adjacent to and apart from adenomas. We found that the expression levels of the 42 upregulated genes were often increased in the mucosae adjacent to the adenomas as well as adenoma themselves (Fig 5D). Indeed, when we scored the samples based on the average expression profiles of the 42 upregulated genes or 5 housekeeping genes, the score of the 42 genes, but not that of housekeeping genes, were elevated in normal mucosae adjacent to adenomas, as well as adenoma themselves, compared with normal mucosae apart from adenomas (S5 Fig). This suggests that, in human patients, the normal epithelial cells adjacent to adenomas have already acquired some traits of human adenoma, as in the case of mouse FP^lo^ cells. However, in the same samples, expression of the 18 commonly downregulated genes was not decreased significantly in the mucosae adjacent to the adenomas (Fig 5D and S5 Fig). This might be due to the fact that human samples collected from the normal mucosae adjacent to adenomas include heterogeneous cell populations. Thus, the decreases in the expression levels of the 18 genes in the population corresponding to FP^lo^ cells might be concealed by the expression signals from other populations. We think that this is also the reason why we could not detect any changes in global gene expression patterns between the normal mucosae adjacent to and apart from adenomas in our microarray analyses (Fig 5B).

### Administration of a DNA methyltransferase inhibitor reverts the silencing of FPs and the aging-associated gene expression alterations

**Fig 6:**
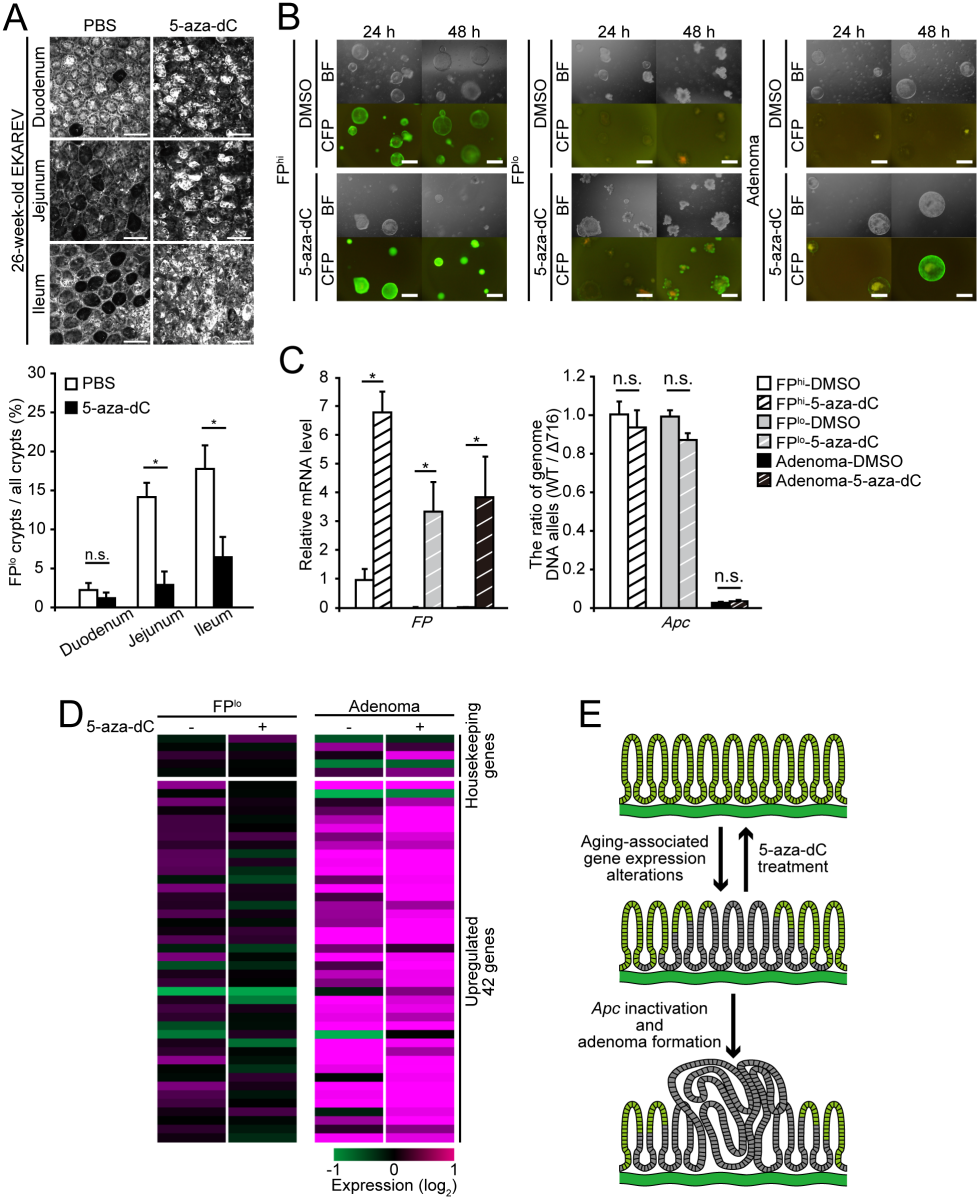
Inhibition of DNA methyltransferases reverts the aging-associated silencing of FPs and gene expression alterations in FP^lo^ cells. (A) (upper) Representative images showing FP expression in the small intestine of 26-week-old EKAREV mice to which 5-aza-dC or vehicle (PBS) was administered. Scale bars, 100 μm. (lower) Quantification of the FP^lo^ cells. *P < 0.05; n.s., not significant (Student’s *t*-test). (B) Representative photographs of *in vitro* cultured organoids derived from FP^hi^, FP^lo^, or adenoma cells. The organoids were treated with DMSO or 5-aza-dC for 24 or 48 hours prior to the imaging. BF, bright field. Scale bars, 100 μm. (C) Relative mRNA expression levels of the EKAREV biosensor (*FP*, left) and the ratio of *Apc*^*WT*^ alleles to *Apc*^*Δ716*^ alleles in FP^hi^, FP^lo^, and adenoma organoids (right). The organoids were treated with DMSO or 5-aza-dC for 48 hours prior to the measurement. *P < 0.05; n.s., not significant (Student’s *t*-test). (D) Quantitative RT-PCR analysis of 5 housekeeping genes, and the 42 upregulated genes, which were identified in Fig 5C, in cultured FP^lo^ and adenoma organoids treated with DMSO or 5-aza-dC for 48 hours. Note that administration of 5-aza-dC suppressed expression of the 42 upregulated genes in FP^lo^ organoids but not in adenoma organoids. (E) Schematic representation of the progression from normal intestinal mucosa to an adenoma.

Finally, we examined the effects of a DNA methyltransferase inhibitor, 5-aza-2’-deoxycitidine (5-aza-dC), on the silencing of FPs and the gene expression alterations during aging, as the CAG promoter, which drives expression of FPs, was highly methylated during aging (Fig 3B). In the small intestine of EKAREV mice, the incidence of FP^lo^ crypts during aging was markedly suppressed by administration of 5-aza-dC (Fig 6A). Similarly to this *in vivo* experiment, administration of 5-aza-dC restored expression of FPs in organoids composed of FP^lo^ or adenoma cells to the levels comparable to those of FP^hi^ crypts (Fig 6B and 6C). The administration of 5-aza-dC did not induce any detectable changes in the DNA copy numbers of the wild-type *Apc* allele and *Apc*^*Δ716*^ allele (Fig 6C). We then analyzed whether 5-aza-dC affects expression levels of the 42 genes upregulated both in FP^lo^ and adenoma cells by qRT-PCR analyses. Expression of most of the 42 genes in FP^lo^ cells was suppressed (Fig 6D). Taken together, these results suggest that DNA methylation mediates the silencing of FPs and gene expression alterations during aging. It should be noted that, in adenoma cells, the expression profiles of the 42 genes were not affected significantly by the 5-aza-dC treatment (Fig 6D). Thus, once cells transform into adenoma cells, the aging-associated gene expression alterations might be fixed through a mechanism independent of DNA methylation.

## Discussion

Aging-associated alterations of stem cell functions have been considered a major cause of a variety of cancers [48]. One of the mechanisms for aging-associated stem cell dysfunction has been supposed to be epigenetic dysregulation, where epigenetic alterations might be accumulated in stem cells and stably transmitted to their progeny during aging, thereby leading to dysfunction of stem cells [10]. However, since most of previous studies have focused on the aging-associated dysfunction in the whole tissue, not in each cell, due to technical difficulties of distinguishing aging cells from non-aging ones in living tissues, the molecular entity of the aging-associated alterations has remained elusive. In this study, we observed the intestinal epithelium of several transgenic mouse lines expressing FPs by 2PM. During the observation, we incidentally found that FPs are ubiquitously expressed in the epithelium of young mice whereas their expression is silenced during aging (Fig 1A and 1B). The silencing of FPs was mediated by DNA hypermethylation of the upstream CAG promoters (Fig 3B). Our microarray analysis then revealed that cells with low expression levels of FPs, which are referred as FP^lo^ cells, harbor specific gene expression signatures compared to cells with high expression levels of FPs, FP^hi^ cells (Fig 4B). Notably, only a small subset of genes (735 genes, about 3% of the genome), showed altered expression between FP^hi^ and FP^lo^ cells. We think that some aging-associated alterations, which cause the observed changes in gene expression, might also induce the silencing of the CAG promoter. Therefore, the silencing of FPs reflects only a specific fraction of aging-associated changes, and if other indicatives for aging are used for analyses, they might be associated with different sets of gene expression changes. An important question is whether the silencing of FPs is stochastic or determined. Given that the silencing of FPs was associated only with specific gene expression signatures, it might be determined or (if it is still stochastic) biased for the silencing. In any case, our results show that the silencing of FPs reflects specific gene expression alterations that occur during aging and can be used as a marker to visualize the aging-associated gene expression alterations in living tissues. Indeed, by utilizing this property of FPs, we could successfully visualize how the aging-associated gene expression alterations spread in each region of the intestinal epithelium during aging (Fig 1B, 1D and 1E). Interestingly, the aging-associated silencing of FPs progressively occurred in units of single crypt; each crypt was almost exclusively composed of either FP^lo^ or FP^hi^ cells, but not of both types of cells (Fig 1B, 1D and 1E). Given that single crypt is composed of clonal progeny of a stem cell, this observation suggests that the aging-associated alterations occur in a stem cell and are stably transmitted to its progeny comprising the crypt. This notion is consistent with a previous report that age-related DNA methylation shows substantial variation between crypts rather than within crypts [49].

While analyzing distribution of FPs, we found that the silencing of FPs always occurs in intestinal adenomas and the surrounding normal mucosae of *Apc*-mutant (*Apc*^*Δ716*^) mice (Fig 2A–2E). Moreover, in these mice, the increase in the number of FP^lo^ cells during aging and/or along the duodenum-ileum axis correlated well with the increased number of adenomas (Figs 1C, 1G and 2G). Collectively, these observations suggest that intestinal adenomas of *Apc*^*Δ716*^ mice originate from the population of FP^lo^ cells. This was supported by further analyses: first, we showed that FP^lo^ cells still retain the wild-type *Apc* allele (Fig 3D–3G), and the epithelium composed of FP^lo^ cells is histologically normal and indistinguishable from that of FP^hi^ cells (Fig 1H). Second, our microarray analyses showed that adenoma cells harbor the gene expression signatures accompanying the aging-associated silencing of FPs (Fig 4C). These findings are consistent with the idea that the aging-associated gene expression alterations, which could be recognized by the silencing of FPs in our experimental settings, precede development of intestinal adenomas in *Apc*-mutant mice. We think that this notion could be applied not only to mouse intestinal adenomas but also human ones. Indeed, we showed that human sporadic and FAP adenomas harbor, at least, a fraction of aging-associated gene expression signatures identified in our mouse experiments. Surprisingly, the gene expression signatures were also observed in the normal mucosae surrounding the FAP adenomas, as in the case of mouse FP^lo^ cells around the adenomas (Fig 5D and S5 Fig). Therefore, it is plausible that the aging-associated gene expression alterations also precede development of intestinal adenomas in human patients (Fig 6E). The aging-associated genes included significantly many ones that are related to cell cycle progression and ones that contribute to development and progression of colorectal tumors, such as *Cd44*, *Myc* and so on (S1 and S2 Tables). It should be clearly described that FP^lo^ cells began to appear at the relatively young age (presumably about 10-week-old) and were increased progressively during aging (we thus described the changes as “the aging-associated signatures”). Therefore, a significant fraction of cells should acquire the aging-associated signatures before a large number of adenoma are formed (about 20-week-old). Given that the tissues harboring the aging-associated gene expression alterations (the tissues composed of FP^lo^ cells) are histologically and functionally normal, these alterations might occur even before the formation of aberrant crypt foci, the earliest preadenomatous regions that are distinguishable from the normal tissues by their abnormal morphology and usually harbor genetic mutations in *KRAS* and/or *p53* in the sporadic cases and in *APC* in the FAP patient cases [50, 51]. Thus, our results suggest that normal intestinal epithelial cells acquire a fraction of tumor-promoting gene expression signatures, which are observed in adenoma cells, during aging before they obtain known genetic mutations. Further analyses of molecular mechanisms and physiological significance of the aging-associated gene expression alterations identified in this study should provide a novel insight into the early processes of the intestinal tumorigenesis.

A key issue yet to be elucidated is how gene expression patterns are changed during aging. Currently, initial cues that trigger gene expression alterations and silencing of FPs are unknown. However, once acquired, the aging-associated gene expression signatures seem to be stably maintained independently of external cues, as FP^lo^ or FP^hi^ cells isolated and cultured *in vitro* as intestinal organoids could stably maintain their respective expression patterns of FPs and gene expression signatures (Fig 6B). In this regard, it should be noted that administration of DNA methyltransferase inhibitor, 5-aza-dC, could revert the silencing of FPs and aging-associated gene expression alterations in FP^lo^ cells (Fig 6A–6D). This suggests that epigenetic regulation by DNA methylation is responsible for the stable maintenance of the aging-associated gene expression signatures and the silencing of FPs. Notably, pharmacological inhibition of DNA methylation has been shown to suppress development of intestinal adenomas in *Apc*-mutant mice [31]. Together with our observations that adenomas develop from FP^lo^ cells, the aging-associated gene expression signatures might play a role in promoting intestinal tumorigenesis. We think that the aging-associated gene expression alterations might confer some growth advantages on *Apc*-mutant cells, thereby promoting development of adenomas. It is also possible that the aging-associated gene expression alterations directly increase the incidence of LOH (or inactivation) of the *Apc* gene, which triggers intestinal tumorigenesis. In any cases, reversion of the aging-associated alterations could be a novel strategy for prevention and treatment of intestinal tumors (Fig 6E).

Because aging is associated with enhanced oxidative stress and inflammation [52], we examined whether we could observe such changes under our experimental condition. However, the urinary F2-isoprostane level (a marker for systemic oxidative stress) [53] or the plasma IL-6 level (a marker for systemic inflammation) [54] was not significantly different between young (20-week-old) and aged (60-week-old) EKAREV mice (S6 and S7 Figs), suggesting that systemic changes in oxidative stress and inflammation were not involved in the observed changes during aging. We also measured the concentration of PGE2 secreted from intestinal epithelial cells, as PGE2 is an important mediator of the inflammation and *Hpgd* (also known as *15-PGDH*), which is involved in the catabolism of PGE2 [55], was downregulated in FP^lo^ and adenoma cells (S3 Table). However, PGE2 was not increased in FP^lo^ cells compared with FP^hi^ cells (S8 Fig). Moreover, the concentration of PGE2 was lowest in adenoma cells (S8 Fig), in which the expression of *Hpgd* was lowest (S9 Fig). Thus, it is not likely that the downregulation of *Hpgd* enhances PGE2 signaling and induces the aging-associated changes in the intestinal epithelium. Notably, downregulation of *Hpgd* in FP^lo^ cells was not abrogated by treatment with the DNA methyltransferase inhibitor (S10 Fig). Therefore, molecular mechanisms of the downregulation of *Hpgd* in FP^lo^ or adenoma cells might be different from those of FPs.

In conclusion, we identified aging-associated gene expression signatures that can be visualized by the silencing of FPs in the intestinal epithelium of several mouse lines, and showed that the signatures precede development of intestinal adenomas in the mouse *Apc*^*Δ716*^ model. The gene expression signatures were also common to the human adenomas and the surrounding normal mucosae, suggesting that similar aging-associated alterations might also occur in human patients. The identification of the aging-associated gene expression signatures should provide a clue for the relationships between aging-associated alterations and intestinal tumorigenesis, and pave the way to development of new drugs for the prevention and treatment of intestinal tumors.

## Supporting Information

S1 Fig The expression patterns of fluorescent proteins in the hematopoietic system. (left) Expression of CFP in leukocytes in young (20-week-old) and aged (60-week-old) EKAREV mice was observed by confocal microscopy. The nuclei of leukocytes were stained with Hoechst (magenta). Note that erythrocytes do not have nuclei, so that cells stained with Hoechst can be recognized as leukocytes. Most of leukocytes from both young and old mice expressed fluorescent proteins (FPs, green). Erythrocytes do not express FPs even in young mice. Scale bars, 25 μm. (right) Quantification of the ratio of leukocytes with or without FP expression (20-week-old: n = 33, 60-week-old: n = 34). n.s., not significant (Student’s *t*-test).
(TIF)

S2 Fig The expression patterns of fluorescent proteins in the skin. Expression of CFP in the skin of a 64-week-old EKAREV mouse was observed by upright 2PM. Two photographs show distinct layers of the skin; the basal layer and suprabasal layer of epidermis. Fluorescent proteins were expressed in all cells in both layers. Scale bars, 100 μm.
(TIF)

S3 Fig Quantification of the EKAREV and the H2B-mCherry transgenes in the R26-H2B-mCherry × EKAREV mouse genome by qPCR. (left) The standard curves were obtained by plotting the relative amount of the target transgenes in 5 standard samples (a dilution series of purified DNA) and the corresponding threshold cycle (Ct) values measured by qPCR (cross marks). Circles and squares indicate duplicated samples from one R26-H2B-mCherry × EKAREV mouse. (right) Quantification of the ratio of the EKAREV transgene to the H2B-mCherry transgene (mean ± SE, n = 2.).
(TIF)

S4 Fig The relative mRNA expression levels of *LGR5* in human samples. The relative mRNA expression levels of *LGR5* obtained from human FAP microarray data (n = 3) (left) or qRT-PCR (n = 8) (right). *P < 0.05; n.s., not significant (Student’s *t*-test).
(TIF)

S5 Fig Average expression profiles of 5 housekeeping genes, 42 upregulated genes, and 18 downregulated genes in human FAP samples. The average expression levels of all genes included in each gene set (housekeeping, upregulated and downregulated genes), relative to controls samples (normal mucosae apart from adenoma), are shown (mean ± SE, n = 8).
(TIF)

S6 Fig Measurement of the urinary F2-isoprostane concentration in young and aged EKAREV mice. (left) The standard curve shown was obtained by plotting the data of a dilution series of purified F2-isoprostane (the concentration of F-2-isoprostane (x-axis-logarithmic) versus the percent absorbance value (y-axis-linear)). Urine samples were collected from young (20-week-old) and aged (60-week-old) EKAREV mice (n = 8 and 6, respectively). (right) Quantification of the results (mean ± SE). n.s., not significant (Student’s *t*-test).
(TIF)

S7 Fig Measurement of the plasma IL-6 concentration in young and aged EKAREV mice. The standard curve shown was generated by plotting the data of a dilution series of the recombinant IL-6 protein (the concentration of IL-6 (x-axis) versus the corresponding absorbance values (OD values) (y-axis)). Plasma samples were collected from young (20-week-old) and aged (60-week-old) EKAREV mice (n = 6 for each).
(TIF)

S8 Fig Measurement of the concentration of PGE2 secreted from intestinal epithelial cells. (left) The standard curve shown was obtained by plotting the data of a dilution series of purified PGE2 (the concentration of PGE2 (x-axis-logarithmic) versus percent absorbance value (y-axis-linear)). Samples were cell supernatants from cultured FP^hi^, FP^lo^ and adenoma cells (n = 3 samples from one 29-week-old *Apc*^*Δ716*^ × EKAREV mouse for each category). (right) Quantification of the results. *P < 0.05; n.s., not significant (Student’s *t*-test).
(TIF)

S9 Fig Relative mRNA expression level of *Hpgd (15-PGDH)* in mouse microarray data. The relative mRNA levels of *Hpgd* obtained from mouse microarray data (Fig 4B) are shown (FP^hi^: n = 4, FP^lo^: n = 6, Adenoma: n = 3).
(TIF)

S10 Fig Treatment with the DNA methyltransferase inhibitor does not abrogate the downregulation of *Hpgd*. The relative mRNA levels of *Hpgd* in FP^lo^ or adenoma cells treated with or without 5-aza-dC were measured by qRT-PCR (FP^lo^: n = 4, Adenoma: n = 3).
(TIF)

S1 Table The top 10 gene ontology terms related to biological processes and overrepresented for the 509 genes upregulated in FP^lo^ crypts. (PDF)

S2 Table The 42 genes commonly upregulated in FP^lo^ crypts, human FAP adenomas and human sporadic adenomas. (PDF)

S3 Table The 18 genes commonly downregulated in FP^lo^ crypts, human FAP adenomas and human sporadic adenomas. (PDF)
