# Supplementary figures and images for: Identification of Aging-Associated Gene Expression Signatures That Precede Intestinal Tumorigenesis
Source: PLoS One. 2016 Sep 2;11(9):e0162300. doi: 10.1371/journal.pone.0162300 (PMC5010213; doi:10.1371/journal.pone.0162300)

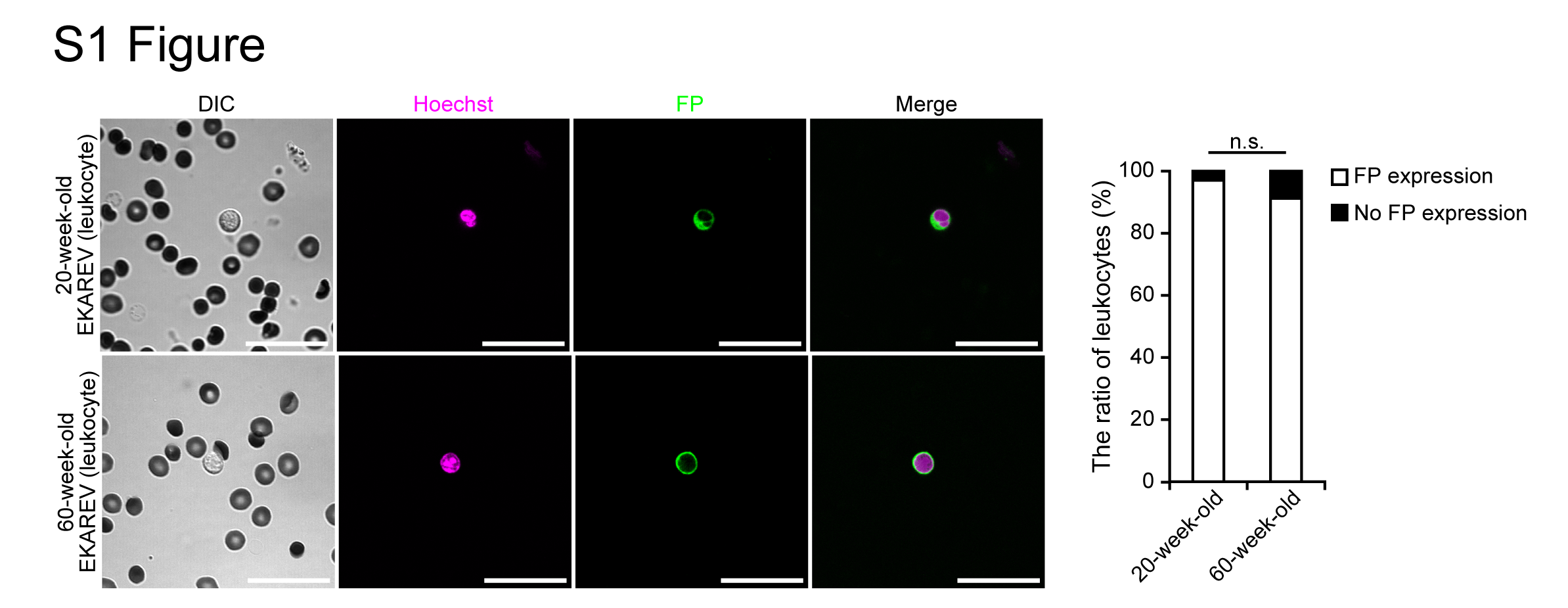

Supplement: S1 Fig — (left) Expression of CFP in leukocytes in young (20-week-old) and aged (60-week-old) EKAREV mice was observed by confocal microscopy. The nuclei of leukocytes were stained with Hoechst (magenta). Note that erythrocytes do not have nuclei, so that cells stained with Hoechst can be recognized as leukocytes. Most of leukocytes from both young and old mice expressed fluorescent proteins (FPs, green). Erythrocytes do not express FPs even in young mice. Scale bars, 25 μm. (right) Quantification of the ratio of leukocytes with or without FP expression (20-week-old: n = 33, 60-week-old: n = 34). n.s., not significant (Student’s t-test). (TIF) [file pone.0162300.s001.tif]

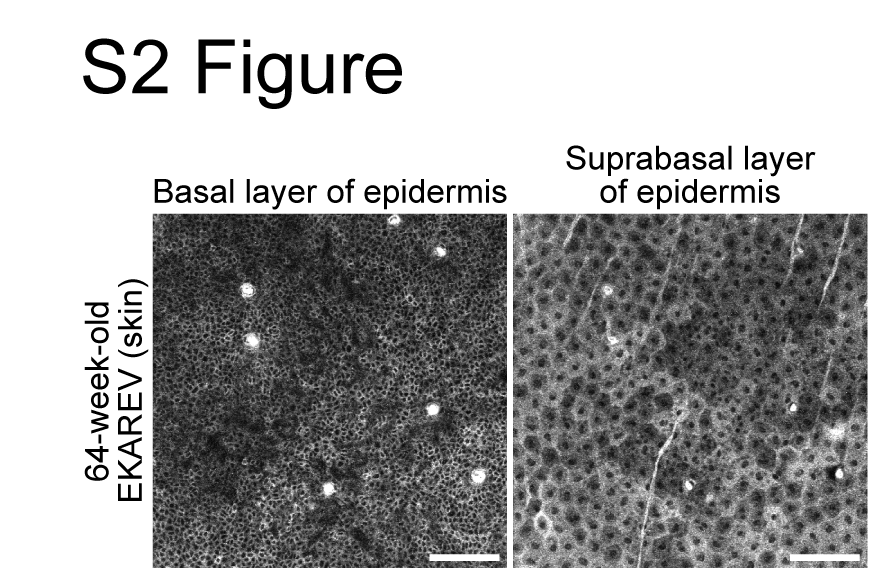

Supplement: S2 Fig — Expression of CFP in the skin of a 64-week-old EKAREV mouse was observed by upright 2PM. Two photographs show distinct layers of the skin; the basal layer and suprabasal layer of epidermis. Fluorescent proteins were expressed in all cells in both layers. Scale bars, 100 μm. (TIF) [file pone.0162300.s002.tif]

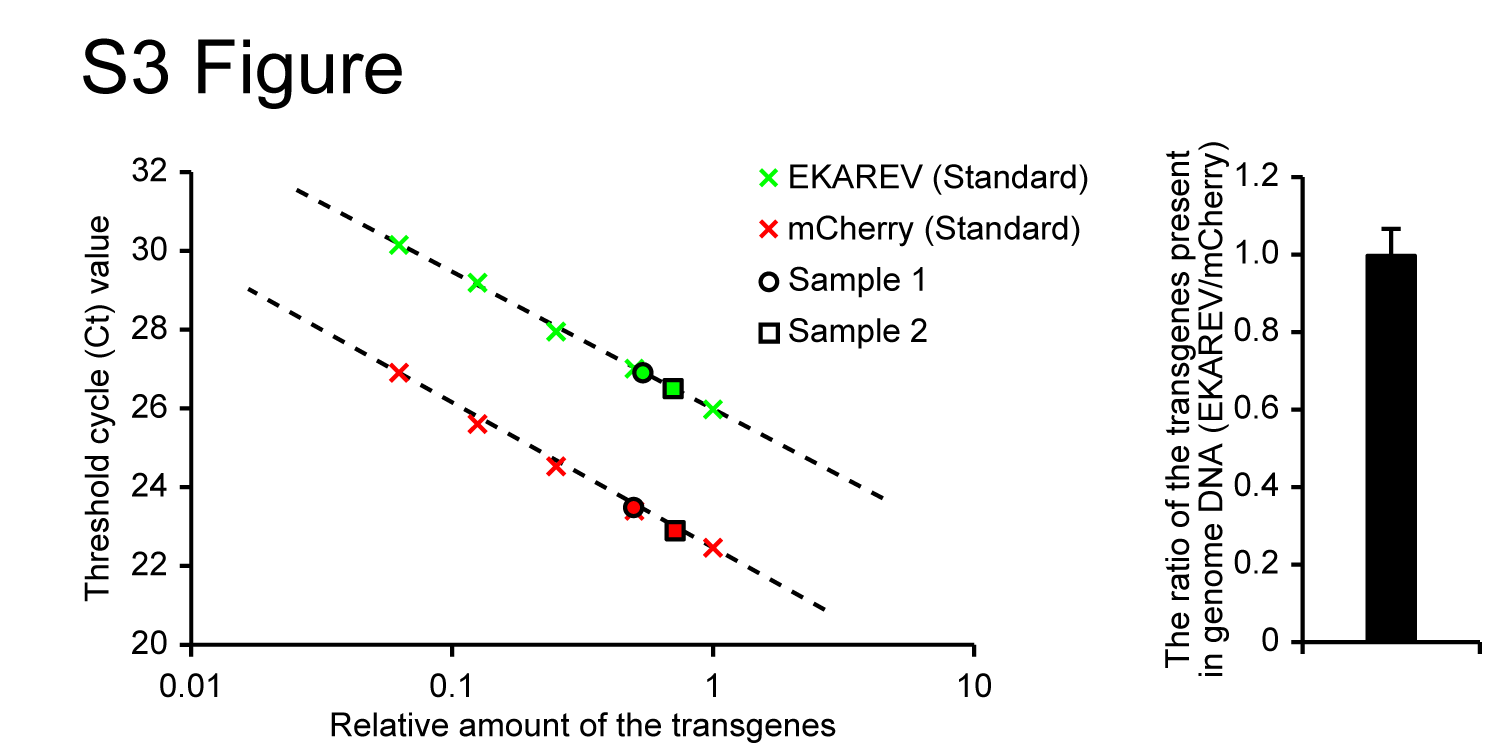

Supplement: S3 Fig — (left) The standard curves were obtained by plotting the relative amount of the target transgenes in 5 standard samples (a dilution series of purified DNA) and the corresponding threshold cycle (Ct) values measured by qPCR (cross marks). Circles and squares indicate duplicated samples from one R26-H2B-mCherry × EKAREV mouse. (right) Quantification of the ratio of the EKAREV transgene to the H2B-mCherry transgene (mean ± SE, n = 2.). (TIF) [file pone.0162300.s003.tif]

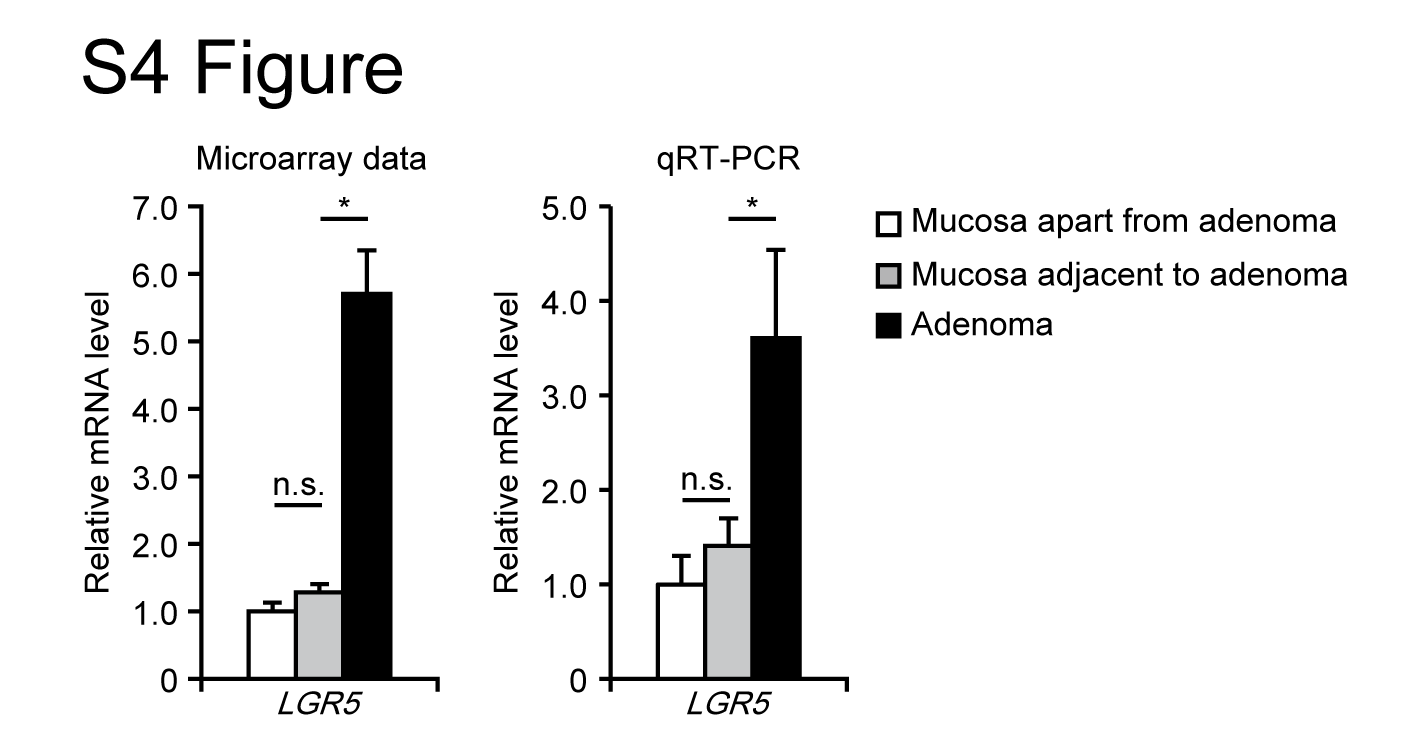

Supplement: S4 Fig — The relative mRNA expression levels of LGR5 obtained from human FAP microarray data (n = 3) (left) or qRT-PCR (n = 8) (right). *P < 0.05; n.s., not significant (Student’s t-test). (TIF) [file pone.0162300.s004.tif]

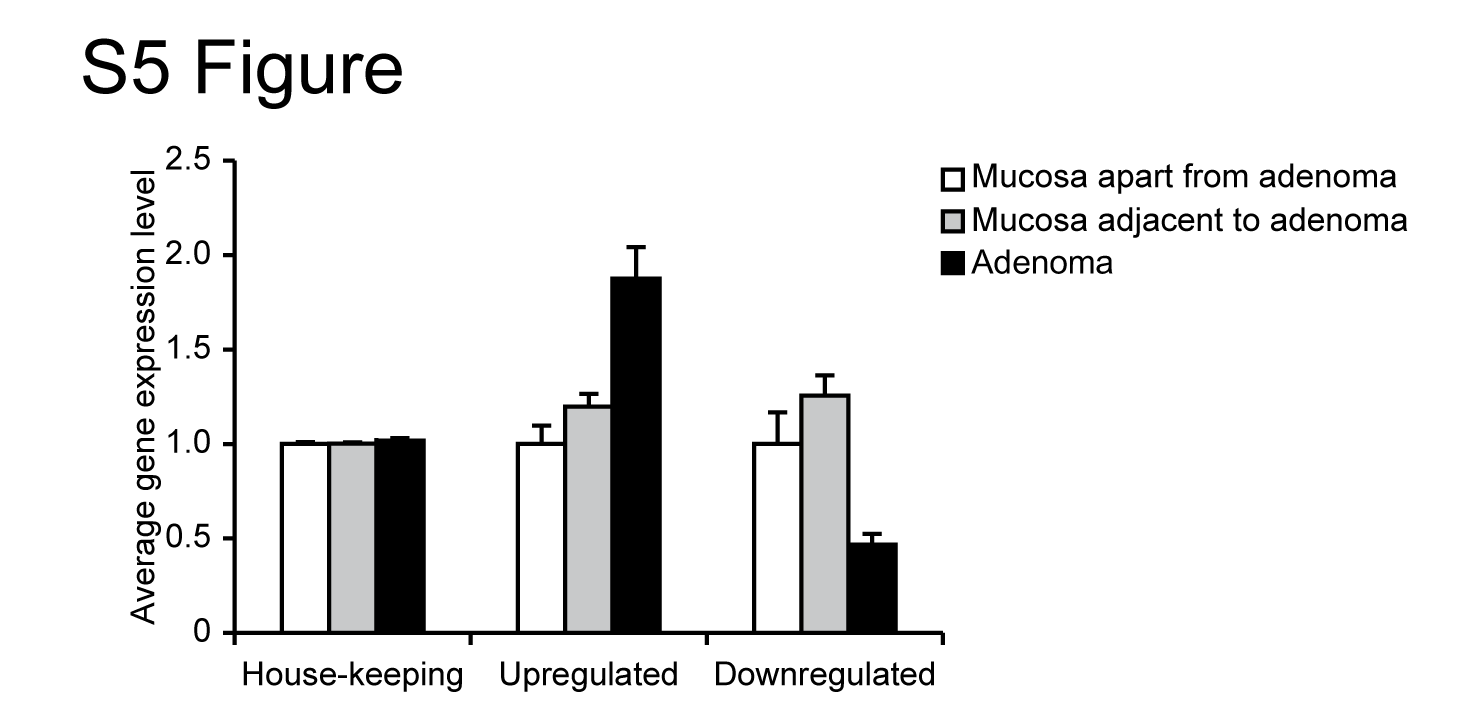

Supplement: S5 Fig — The average expression levels of all genes included in each gene set (housekeeping, upregulated and downregulated genes), relative to controls samples (normal mucosae apart from adenoma), are shown (mean ± SE, n = 8). (TIF) [file pone.0162300.s005.tif]

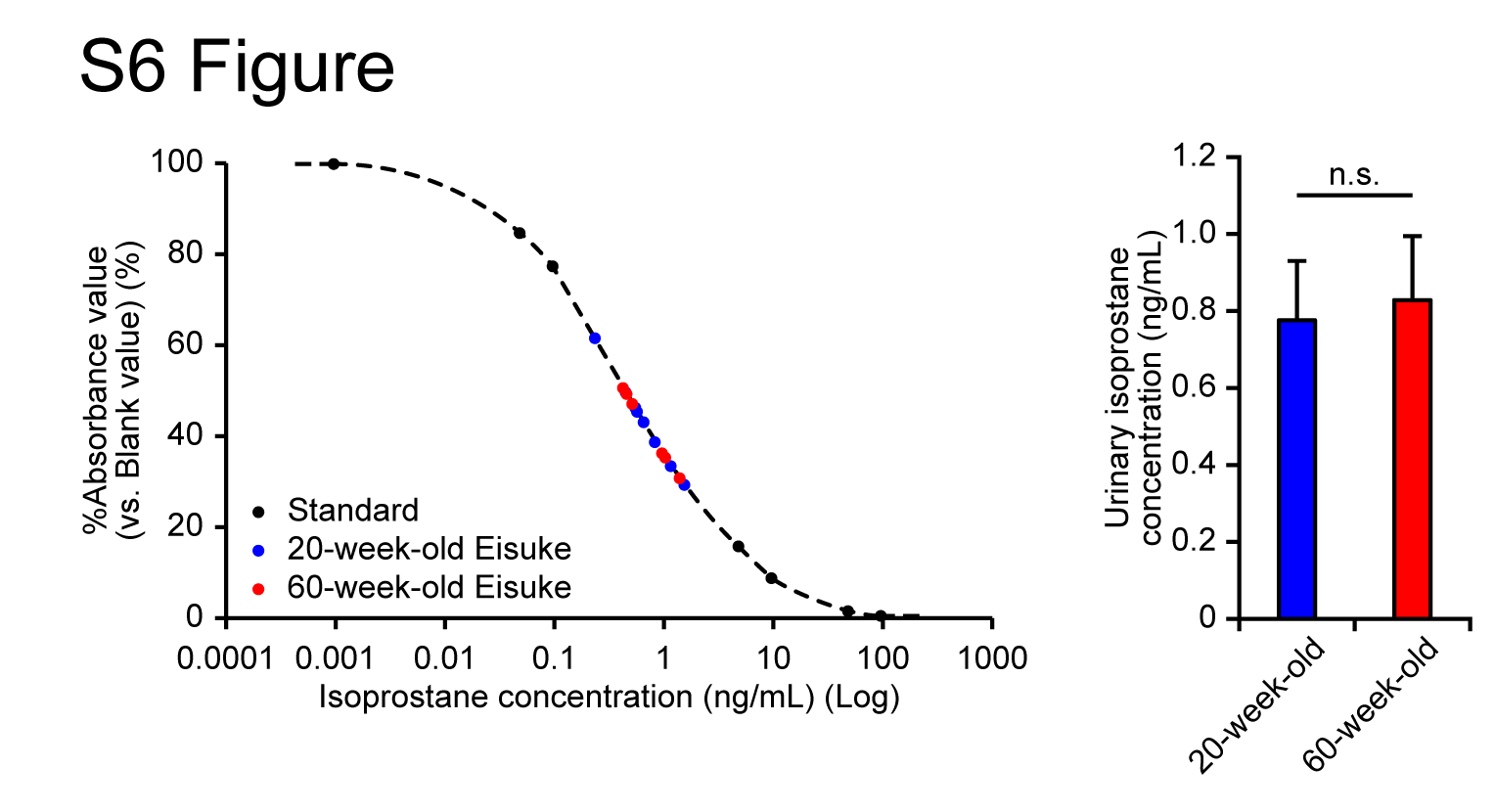

Supplement: S6 Fig — (left) The standard curve shown was obtained by plotting the data of a dilution series of purified F2-isoprostane (the concentration of F-2-isoprostane (x-axis-logarithmic) versus the percent absorbance value (y-axis-linear)). Urine samples were collected from young (20-week-old) and aged (60-week-old) EKAREV mice (n = 8 and 6, respectively). (right) Quantification of the results (mean ± SE). n.s., not significant (Student’s t-test). (TIF) [file pone.0162300.s006.tif]

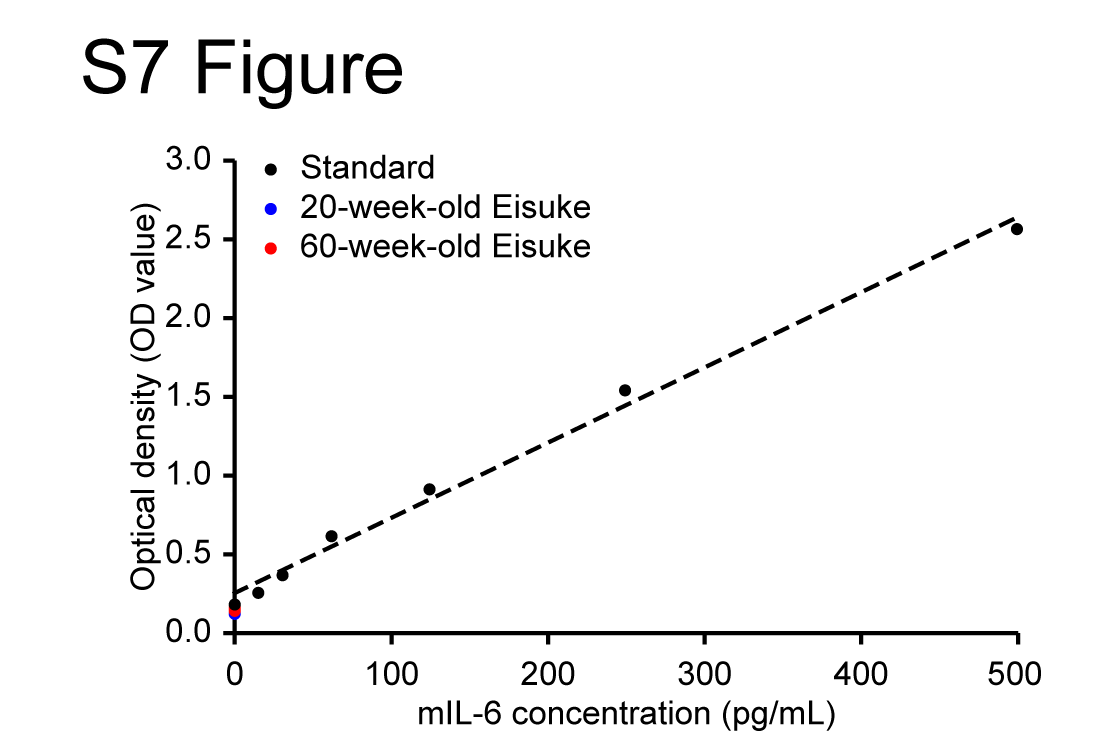

Supplement: S7 Fig — The standard curve shown was generated by plotting the data of a dilution series of the recombinant IL-6 protein (the concentration of IL-6 (x-axis) versus the corresponding absorbance values (OD values) (y-axis)). Plasma samples were collected from young (20-week-old) and aged (60-week-old) EKAREV mice (n = 6 for each). (TIF) [file pone.0162300.s007.tif]

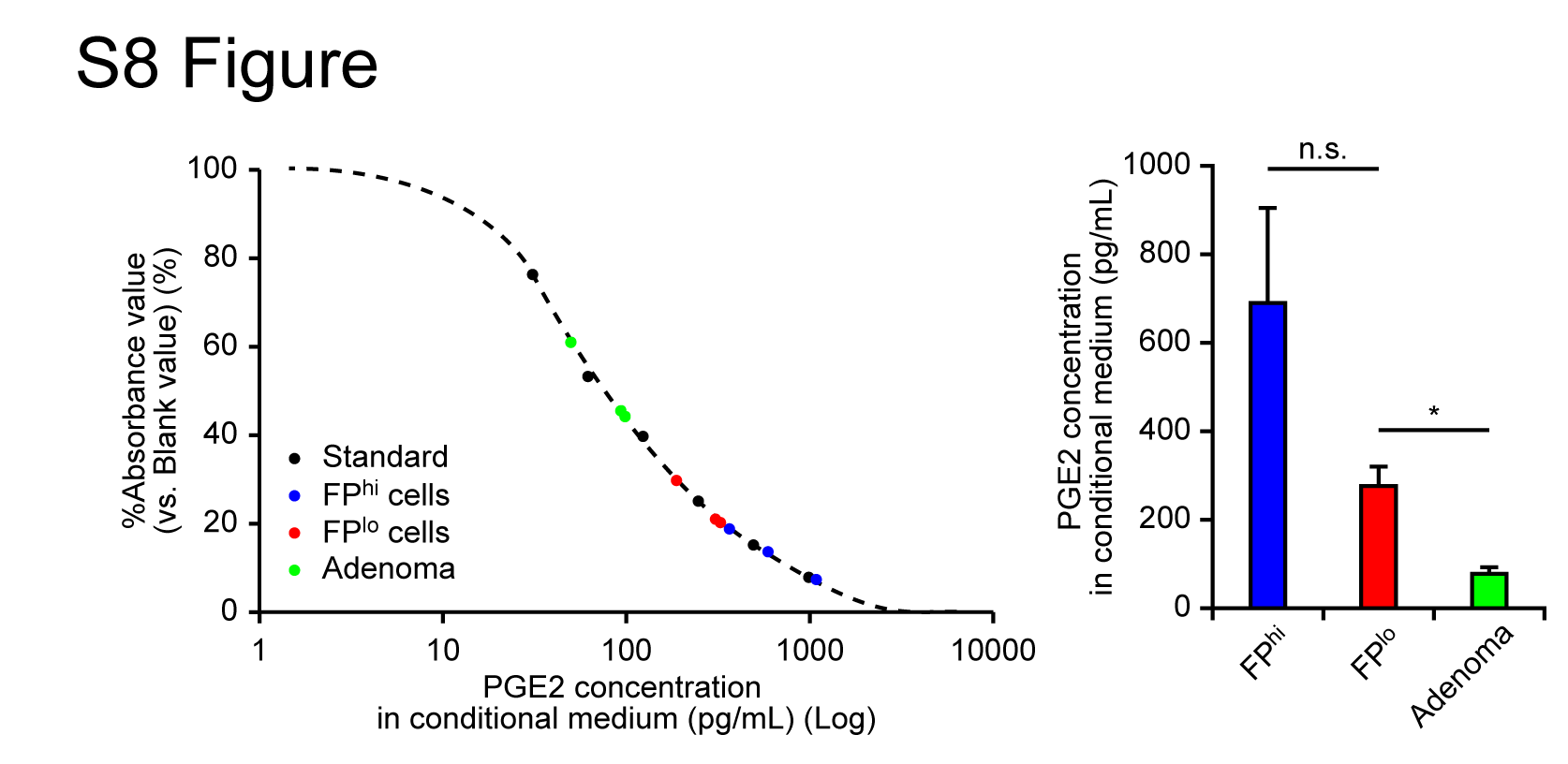

Supplement: S8 Fig — (left) The standard curve shown was obtained by plotting the data of a dilution series of purified PGE2 (the concentration of PGE2 (x-axis-logarithmic) versus percent absorbance value (y-axis-linear)). Samples were cell supernatants from cultured FPhi, FPlo and adenoma cells (n = 3 samples from one 29-week-old ApcΔ716 × EKAREV mouse for each category). (right) Quantification of the results. *P < 0.05; n.s., not significant (Student’s t-test). (TIF) [file pone.0162300.s008.tif]

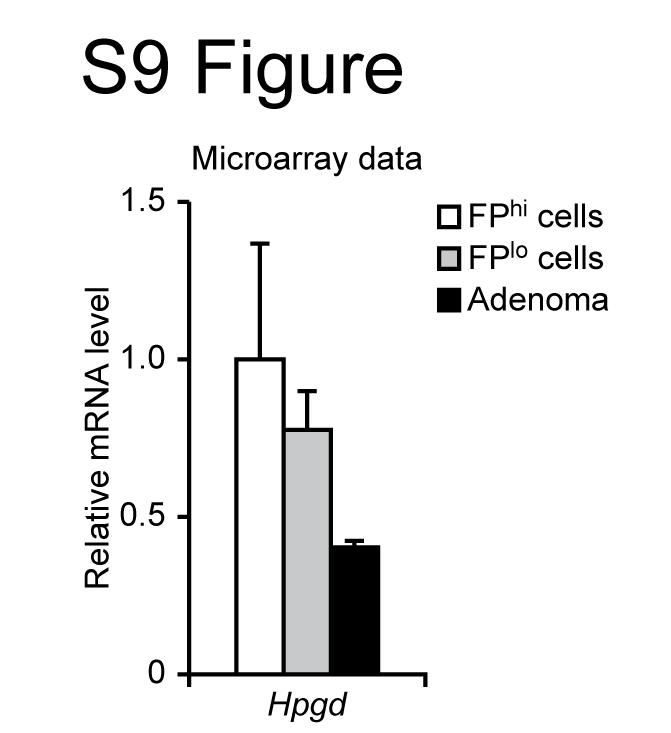

Supplement: S9 Fig — The relative mRNA levels of Hpgd obtained from mouse microarray data (Fig 4B) are shown (FPhi: n = 4, FPlo: n = 6, Adenoma: n = 3). (TIF) [file pone.0162300.s009.tif]

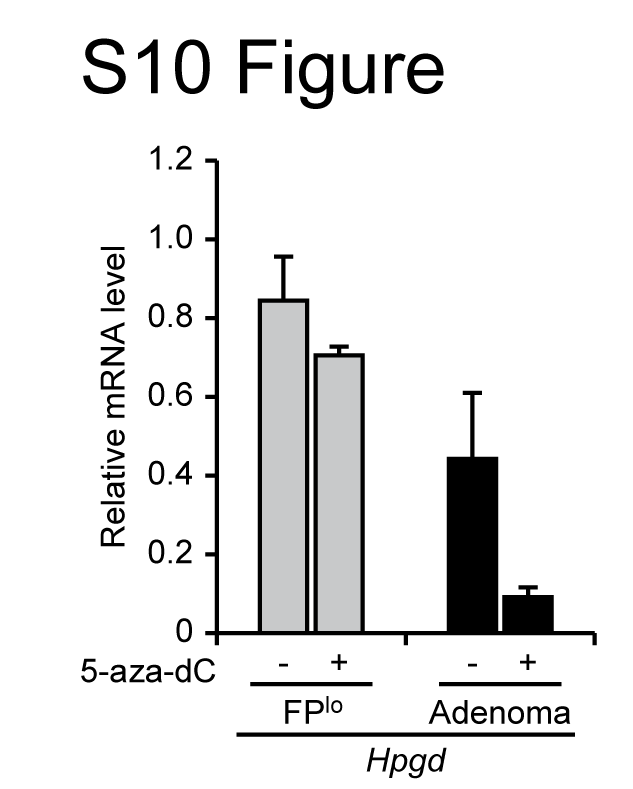

Supplement: S10 Fig — The relative mRNA levels of Hpgd in FPlo or adenoma cells treated with or without 5-aza-dC were measured by qRT-PCR (FPlo: n = 4, Adenoma: n = 3). (TIF) [file pone.0162300.s010.tif]
