# Supplementary material for: Identification of Aging-Associated Gene Expression Signatures That Precede Intestinal Tumorigenesis
Source: PLoS One. 2016 Sep 2;11(9):e0162300. doi: 10.1371/journal.pone.0162300 (PMC5010213; doi:10.1371/journal.pone.0162300)
Supplement: S1 Table — (PDF) [file pone.0162300.s011.pdf]

S1 Table. The top 10 gene ontology terms related to biological processes and overrepresented for the 509 genes upregulated in FP^{lo} crypts.

Term	Count	P-Value
GO:0007049~cell cycle	65	1.34E-20
GO:0022403~cell cycle phase	45	2.20E-18
GO:0000278~mitotic cell cycle	37	1.49E-16
GO:0022402~cell cycle process	46	5.14E-16
GO:0000279~M phase	39	6.12E-16
GO:0051301~cell division	37	1.61E-14
GO:0000087~M phase of mitotic cell cycle	31	1.76E-14
GO:0000280~nuclear division	30	6.93E-14
GO:0007067~mitosis	30	6.93E-14
GO:0048285~organelle fission	30	1.80E-13
