# Supplementary material for: Identification of Aging-Associated Gene Expression Signatures That Precede Intestinal Tumorigenesis
Source: PLoS One. 2016 Sep 2;11(9):e0162300. doi: 10.1371/journal.pone.0162300 (PMC5010213; doi:10.1371/journal.pone.0162300)
Supplement: S2 Table — (PDF) [file pone.0162300.s012.pdf]

S2 Table. The 42 genes commonly upregulated in FP^{lo} crypts, human FAP adenomas and human sporadic adenomas.

Symbol	Name	Fold Change (FP^{lo} vs FP^{hi})	Association with tumor progression and prognosis: CRC	Association with tumor progression and prognosis: Other tumors	Reference
Chek1	checkpoint kinase 1	5.21	+	+	(1,2)
Zdhhc2	zinc finger, DHHC domain containing 2	3.91			
Mlec	malectin	3.46			
Txnl4b	thioredoxin-like 4B	3.40			
Cdk6	cyclin-dependent kinase 6	3.29	+	+	(3,4)
Impdh2	inosine 5'-phosphate dehydrogenase 2	3.17	+	+	(5-7)
Cks2	CDC28 protein kinase regulatory subunit 2	3.11	+	+	(8-11)
H2afz	H2A histone family, member Z	2.93	+	+	(12,13)
Ruvbl1	RuvB-like protein 1	2.77	+	+	(14-16)
Top2a	topoisomerase (DNA) II alpha	2.75	+	+	(17,18)
Rfc3	replication factor C (activator 1) 3	2.65	+	+	(19,20)
Wdr74	WD repeat domain 74	2.63			
Pola1	polymerase (DNA directed), alpha 1	2.60			
Tfb1m	transcription factor B1, mitochondrial	2.60			
Rlim	ring finger protein, LIM domain interacting	2.54			
Cenpk	centromere protein K	2.54		+	(21)
Mki67	antigen identified by monoclonal antibody Ki 67	2.52	+	+	(22)
Cks1b	CDC28 protein kinase 1b	2.45			
Smarcc1	SWI/SNF related, matrix associated, actin dependent regulator of chromatin, subfamily c, member 1	2.42			
Fermt1	fermitin family homolog 1 (Drosophila)	2.41	+	+	(23,24)
Lmnb1	lamin B1	2.40	+	+	(25,26)
Slc7a5	solute carrier family 7 (cationic amino acid transporter, y+ system), member 5	2.39		+	(27)
Nip7	nuclear import 7 homolog (S. cerevisiae)	2.35		+	(28)
Ddx10	DEAD (Asp-Glu-Ala-Asp) box polypeptide 10	2.34			
Ggh	gamma-glutamyl hydrolase	2.31	+	+	(29-31)
Cd44	CD44 antigen	2.31	+	+	(32,33)
Ssrp1	structure specific recognition protein 1	2.30			
Cse1l	chromosome segregation 1-like (S. cerevisiae)	2.27	+	+	(34-36)
Kcne3	potassium voltage-gated channel, Isk-related subfamily, gene 3	2.26			
Pgap1	post-GPI attachment to proteins 1	2.19			
Apip	APAF1 interacting protein	2.19		+	(37)
Srpk1	serine/arginine-rich protein specific kinase 1	2.13	+	+	(38,39)
Nup85	nucleoporin 85	2.12			
Wdr75	WD repeat domain 75	2.12			
Nup88	nucleoporin 88	2.11	+	+	(40-42)

Hells	helicase, lymphoid specific	2.11		+	(43)
Exosc2	exosome component 2	2.11			
Myc	myelocytomatosis oncogene	2.07	+	+	(44,45)
Trap1	TNF receptor-associated protein 1	2.06	+	+	(46,47)
Wdr12	WD repeat domain 12	2.06			
Trit1	tRNA isopentenyltransferase 1	2.04			
Ebna1bp2	EBNA1 binding protein 2	2.03		+	(48)

Supporting Reference

1. Gali-Muhtasib H, Kuester D, Mawrin C, Bajbouj K, Diestel A, Ocker M, et al. Thymoquinone triggers inactivation of the stress response pathway sensor CHEK1 and contributes to apoptosis in colorectal cancer cells. Cancer Res 2008;68(14):5609-18.
2. Mohni KN, Kavanaugh GM, Cortez D. ATR pathway inhibition is synthetically lethal in cancer cells with ERCC1 deficiency. Cancer Res 2014;74(10):2835-45.
3. Salh B, Bergman D, Marotta A, Pelech SL. Differential cyclin-dependent kinase expression and activation in human colon cancer. Anticancer research 1999;19(1B):741-8.
4. Sherr CJ, Beach D, Shapiro GI. Targeting CDK4 and CDK6: From Discovery to Therapy. Cancer discovery 2015.
5. He Y, Mou Z, Li W, Liu B, Fu T, Zhao S, et al. Identification of IMPDH2 as a tumor-associated antigen in colorectal cancer using immunoproteomics analysis. International journal of colorectal disease 2009;24(11):1271-9.
6. Tunca B, Tezcan G, Cecener G, Egeli U, Zorluoglu A, Yilmazlar T, et al. Overexpression of CK20, MAP3K8 and EIF5A correlates with poor prognosis in early-onset colorectal cancer patients. Journal of cancer research and clinical oncology 2013;139(4):691-702.
7. Zhou L, Xia D, Zhu J, Chen Y, Chen G, Mo R, et al. Enhanced expression of IMPDH2 promotes metastasis and advanced tumor progression in patients with prostate cancer. Clinical & translational oncology : official publication of the Federation of Spanish Oncology Societies and of the National Cancer Institute of Mexico 2014;16(10):906-13.
8. Lin HM, Chatterjee A, Lin YH, Anjomshoaa A, Fukuzawa R, McCall JL, et al. Genome wide expression profiling identifies genes associated with colorectal liver metastasis. Oncology reports 2007;17(6):1541-9.
9. Jung Y, Lee S, Choi HS, Kim SN, Lee E, Shin Y, et al. Clinical validation of colorectal cancer biomarkers identified from bioinformatics analysis of public expression data. Clinical cancer research : an official journal of the American Association for Cancer Research 2011;17(4):700-9.

10. Yu MH, Luo Y, Qin SL, Wang ZS, Mu YF, Zhong M. Up-regulated CKS2 promotes tumor progression and predicts a poor prognosis in human colorectal cancer. American journal of cancer research 2015;5(9):2708-18.
11. You H, Lin H, Zhang Z. CKS2 in human cancers: Clinical roles and current perspectives (Review). Molecular and clinical oncology 2015;3(3):459-63.
12. Wang JY, Wang YH, Jao SW, Lu CY, Kuo CH, Hu HM, et al. Molecular mechanisms underlying the tumorigenesis of colorectal adenomas: correlation to activated K-ras oncogene. Oncology reports 2006;16(6):1245-52.
13. Yang HD, Kim PJ, Eun JW, Shen Q, Kim HS, Shin WC, et al. Oncogenic potential of histone-variant H2A.Z.1 and its regulatory role in cell cycle and epithelial-mesenchymal transition in liver cancer. Oncotarget 2016.
14. Lauscher JC, Loddenkemper C, Kosel L, Grone J, Buhr HJ, Huber O. Increased pontin expression in human colorectal cancer tissue. Human pathology 2007;38(7):978-85.
15. Lauscher JC, Elezkurtaj S, Dullat S, Lipka S, Grone J, Buhr HJ, et al. Increased Pontin expression is a potential predictor for outcome in sporadic colorectal carcinoma. Oncology reports 2012;28(5):1619-24.
16. Grigoletto A, Lestienne P, Rosenbaum J. The multifaceted proteins Reptin and Pontin as major players in cancer. Biochimica et biophysica acta 2011;1815(2):147-57.
17. Al-Kuraya K, Novotny H, Bavi P, Siraj AK, Uddin S, Ezzat A, et al. HER2, TOP2A, CCND1, EGFR and C-MYC oncogene amplification in colorectal cancer. Journal of clinical pathology 2007;60(7):768-72.
18. Reinholz MM, Bruzek AK, Visscher DW, Lingle WL, Schroeder MJ, Perez EA, et al. Breast cancer and aneusomy 17: implications for carcinogenesis and therapeutic response. The Lancet Oncology 2009;10(3):267-77.
19. Kim YR, Song SY, Kim SS, An CH, Lee SH, Yoo NJ. Mutational and expressional analysis of RFC3, a clamp loader in DNA replication, in gastric and colorectal cancers. Human pathology 2010;41(10):1431-7.
20. Shen H, Xu J, Zhao S, Shi H, Yao S, Jiang N. ShRNA-mediated silencing of the RFC3 gene suppress ovarian tumor cells proliferation. International journal of clinical and experimental pathology 2015;8(8):8968-75.
21. Lee YC, Huang CC, Lin DY, Chang WC, Lee KH. Overexpression of centromere protein K (CENPK) in ovarian cancer is correlated with poor patient survival and associated with predictive and prognostic

relevance. PeerJ 2015;3:e1386.

22. Li LT, Jiang G, Chen Q, Zheng JN. Ki67 is a promising molecular target in the diagnosis of cancer (review). Molecular medicine reports 2015;11(3):1566-72.

23. Fan J, Yan D, Teng M, Tang H, Zhou C, Wang X, et al. Digital transcript profile analysis with aRNA-LongSAGE validates FERMT1 as a potential novel prognostic marker for colon cancer. Clinical cancer research : an official journal of the American Association for Cancer Research 2011;17(9):2908-18.

24. Culhane AC, Quackenbush J. Confounding effects in "A six-gene signature predicting breast cancer lung metastasis". Cancer Res 2009;69(18):7480-5.

25. Marshall KW, Mohr S, Khettabi FE, Nossova N, Chao S, Bao W, et al. A blood-based biomarker panel for stratifying current risk for colorectal cancer. International journal of cancer Journal international du cancer 2010;126(5):1177-86.

26. Sun S, Xu MZ, Poon RT, Day PJ, Luk JM. Circulating Lamin B1 (LMNB1) biomarker detects early stages of liver cancer in patients. Journal of proteome research 2010;9(1):70-8.

27. Bhutia YD, Babu E, Ramachandran S, Ganapathy V. Amino Acid transporters in cancer and their relevance to "glutamine addiction": novel targets for the design of a new class of anticancer drugs. Cancer Res 2015;75(9):1782-8.

28. Savci-Heijink CD, Halfwerk H, Koster J, van de Vijver MJ. A novel gene expression signature for bone metastasis in breast carcinomas. Breast cancer research and treatment 2016.

29. Odin E, Wettergren Y, Nilsson S, Willen R, Carlsson G, Spears CP, et al. Altered gene expression of folate enzymes in adjacent mucosa is associated with outcome of colorectal cancer patients. Clinical cancer research : an official journal of the American Association for Cancer Research 2003;9(16 Pt 1):6012-9.

30. Kidd EA, Yu J, Li X, Shannon WD, Watson MA, McLeod HL. Variance in the expression of 5-Fluorouracil pathway genes in colorectal cancer. Clinical cancer research : an official journal of the American Association for Cancer Research 2005;11(7):2612-9.

31. Shubbar E, Helou K, Kovacs A, Nemes S, Hajizadeh S, Enerback C, et al. High levels of gamma-glutamyl hydrolase (GGH) are associated with poor prognosis and unfavorable clinical outcomes in invasive breast cancer. BMC cancer 2013;13:47.

32. Du L, Wang H, He L, Zhang J, Ni B, Wang X, et al. CD44 is of functional importance for colorectal cancer stem cells. Clinical cancer research : an official journal of the American Association for Cancer Research 2008;14(21):6751-60.

33. Yan Y, Zuo X, Wei D. Concise Review: Emerging Role of CD44 in Cancer Stem Cells: A Promising

Biomarker and Therapeutic Target. Stem cells translational medicine 2015;4(9):1033-43.

34. Tsao TY, Tsai CS, Tung JN, Chen SL, Yue CH, Liao CF, et al. Function of CSE1L/CAS in the secretion of HT-29 human colorectal cells and its expression in human colon. Molecular and cellular biochemistry 2009;327(1-2):163-70.

35. Alnabulsi A, Agouni A, Mitra S, Garcia-Murillas I, Carpenter B, Bird S, et al. Cellular apoptosis susceptibility (chromosome segregation 1-like, CSE1L) gene is a key regulator of apoptosis, migration and invasion in colorectal cancer. J Pathol 2012;228(4):471-81.

36. Wellmann A, Flemming P, Behrens P, Wuppermann K, Lang H, Oldhafer K, et al. High expression of the proliferation and apoptosis associated CSE1L/CAS gene in hepatitis and liver neoplasms: correlation with tumor progression. International journal of molecular medicine 2001;7(5):489-94.

37. Hong SH, Lee WJ, Kim YD, Kim H, Jeon YJ, Lim B, et al. APIP, an ERBB3-binding partner, stimulates erbB2-3 heterodimer formation to promote tumorigenesis. Oncotarget 2016.

38. Hayes GM, Carrigan PE, Miller LJ. Serine-arginine protein kinase 1 overexpression is associated with tumorigenic imbalance in mitogen-activated protein kinase pathways in breast, colonic, and pancreatic carcinomas. Cancer Res 2007;67(5):2072-80.

39. Hayes GM, Carrigan PE, Beck AM, Miller LJ. Targeting the RNA splicing machinery as a novel treatment strategy for pancreatic carcinoma. Cancer Res 2006;66(7):3819-27.

40. Emterling A, Skoglund J, Arbman G, Schneider J, Evertsson S, Carstensen J, et al. Clinicopathological significance of Nup88 expression in patients with colorectal cancer. Oncology 2003;64(4):361-9.

41. Zhang ZY, Zhao ZR, Jiang L, Li JC, Gao YM, Cui DS, et al. Nup88 expression in normal mucosa, adenoma, primary adenocarcinoma and lymph node metastasis in the colorectum. Tumour biology : the journal of the International Society for Oncodevelopmental Biology and Medicine 2007;28(2):93-9.

42. Agudo D, Gomez-Esquer F, Martinez-Arribas F, Nunez-Villar MJ, Pollan M, Schneider J. Nup88 mRNA overexpression is associated with high aggressiveness of breast cancer. International journal of cancer Journal international du cancer 2004;109(5):717-20.

43. Colak D, Nofal A, Albakheet A, Nirmal M, Jeprel H, Eldali A, et al. Age-specific gene expression signatures for breast tumors and cross-species conserved potential cancer progression markers in young women. PloS one 2013;8(5):e63204.

44. Wilkins JA, Sansom OJ. C-Myc is a critical mediator of the phenotypes of Apc loss in the intestine. Cancer Res 2008;68(13):4963-6.

45. Stine ZE, Walton ZE, Altman BJ, Hsieh AL, Dang CV. MYC, Metabolism, and Cancer. Cancer

discovery 2015;5(10):1024-39.

46. Chen R, Pan S, Lai K, Lai LA, Crispin DA, Bronner MP, et al. Up-regulation of mitochondrial chaperone TRAP1 in ulcerative colitis associated colorectal cancer. World J Gastroenterol 2014;20(45):17037-48.

47. Zhang B, Wang J, Huang Z, Wei P, Liu Y, Hao J, et al. Aberrantly upregulated TRAP1 is required for tumorigenesis of breast cancer. Oncotarget 2015;6(42):44495-508.

48. Pilarsky C, Wenzig M, Specht T, Saeger HD, Grutzmann R. Identification and validation of commonly overexpressed genes in solid tumors by comparison of microarray data. Neoplasia 2004;6(6):744-50.
