# Supplementary material for: Identification of Aging-Associated Gene Expression Signatures That Precede Intestinal Tumorigenesis
Source: PLoS One. 2016 Sep 2;11(9):e0162300. doi: 10.1371/journal.pone.0162300 (PMC5010213; doi:10.1371/journal.pone.0162300)
Supplement: S3 Table — (PDF) [file pone.0162300.s013.pdf]

S3 Table. The 18 genes commonly downregulated in FP^lo crypts, human FAP adenomas and human sporadic adenomas.

Symbol	Name	Fold Change (FP^lo vs. FP^hi)	Association with tumor progression and prognosis		Reference
			CRC	Other tumors	
Plac8	placenta-specific 8	-1.53			
Arhgef6	Rac/Cdc42 guanine nucleotide exchange factor (GEF) 6	-1.55			
Hpgd	hydroxyprostaglandin dehydrogenase 15 (NAD)	-1.57	-		(1)
Mall	mal, T cell differentiation protein-like	-1.63			
Pls1	plastin 1 (I-isoform)	-1.67			
Mep1a	meprin 1 alpha	-1.71			
Ctss	cathepsin S	-1.72			
Zg16	zymogen granule protein 16	-1.74		-	(2)
Mxd1	MAX dimerization protein 1	-1.75		-	(3)
Ppap2a	phosphatidic acid phosphatase type 2A	-1.77			
Ccdc68	coiled-coil domain containing 68	-1.8	-	-	(4,5)
Xdh	xanthine dehydrogenase	-1.8			
Dhrs11	dehydrogenase/reductase (SDR family) member 11	-1.95			
Sepp1	selenoprotein P, plasma, 1	-2.14	-		(6)
Hsd17b2	hydroxysteroid (17-beta) dehydrogenase 2	-2.31			
Papss2	3'-phosphoadenosine 5'-phosphosulfate synthase 2	-2.4			
Slc26a3	solute carrier family 26, member 3	-3.02	-	-	(7,8)
Krt20	keratin 20	-4.85			

Supporting Reference

1. Fink SP, Yamauchi M, Nishihara R, Jung S, Kuchiba A, Wu K, et al. Aspirin and the risk of colorectal cancer in relation to the expression of 15-hydroxyprostaglandin dehydrogenase (HPGD). Science translational medicine 2014;6(233):233re2.
2. Zhou YB, Cao JB, Yang HM, Zhu H, Xu ZG, Wang KS, et al. hZG16, a novel human secreted protein expressed in liver, was down-regulated in hepatocellular carcinoma. Biochemical and biophysical research communications 2007;355(3):679-86.
3. O'Hagan RC, Schreiber-Agus N, Chen K, David G, Engelman JA, Schwab R, et al. Gene-target recognition among members of the myc superfamily and implications for oncogenesis. Nat Genet 2000;24(2):113-9.
4. Sheffer M, Bacolod MD, Zuk O, Giardina SF, Pincas H, Barany F, et al. Association of survival and disease progression with chromosomal instability: a genomic exploration of colorectal cancer. Proc

Natl Acad Sci U S A 2009;106(17):7131-6.

5. Radulovich N, Leung L, Ibrahimov E, Navab R, Sakashita S, Zhu CQ, et al. Coiled-coil domain containing 68 (CCDC68) demonstrates a tumor-suppressive role in pancreatic ductal adenocarcinoma. Oncogene 2015;34(32):4238-47.
6. Barrett CW, Reddy VK, Short SP, Motley AK, Lintel MK, Bradley AM, et al. Selenoprotein P influences colitis-induced tumorigenesis by mediating stemness and oxidative damage. J Clin Invest 2015;125(7):2646-60.
7. Mlakar V, Berginc G, Volavsek M, Stor Z, Rems M, Glavac D. Presence of activating KRAS mutations correlates significantly with expression of tumour suppressor genes DCN and TPM1 in colorectal cancer. BMC cancer 2009;9:282.
8. Di Stadio CS, Altieri F, Miselli G, Elce A, Severino V, Chambery A, et al. AMP18 interacts with the anion exchanger SLC26A3 and enhances its expression in gastric cancer cells. Biochimie 2016;121:151-60.
